# Autophosphorylation of the Bacterial Tyrosine-Kinase CpsD Connects Capsule Synthesis with the Cell Cycle in *Streptococcus pneumoniae*

**DOI:** 10.1371/journal.pgen.1005518

**Published:** 2015-09-17

**Authors:** Julien Nourikyan, Morten Kjos, Chryslène Mercy, Caroline Cluzel, Cécile Morlot, Marie-Francoise Noirot-Gros, Sébastien Guiral, Jean-Pierre Lavergne, Jan-Willem Veening, Christophe Grangeasse

**Affiliations:** 1 Bases Moléculaires et Structurales des Systèmes Infectieux, UMR5086 CNRS/Université de Lyon 1, Lyon, France; 2 Molecular Genetics Group, Groningen Biomolecular Sciences and Biotechnology Institute, Centre for Synthetic Biology, University of Groningen, Groningen, the Netherlands; 3 Laboratoire Biologie Tissulaire et Ingénierie thérapeutique, UMR5305, CNRS/Université de Lyon 1, Lyon, France; 4 Institut de Biologie Structurale, UMR5075 CNRS/CEA/Université Grenoble Alpes, Grenoble, France; 5 Micalis, UMR1319 INRA, Jouy-en-Josas, France; University of Geneva Medical School, SWITZERLAND

## Abstract

Bacterial capsular polysaccharides (CPS) are produced by a multi-protein membrane complex, in which a particular type of tyrosine-autokinases named BY-kinases, regulate their polymerization and export. However, our understanding of the role of BY-kinases in these processes remains incomplete. In the human pathogen *Streptococcus pneumoniae*, the BY-kinase CpsD localizes at the division site and participates in the proper assembly of the capsule. In this study, we show that the cytoplasmic C-terminal end of the transmembrane protein CpsC is required for CpsD autophosphorylation and localization at mid-cell. Importantly, we demonstrate that the CpsC/CpsD complex captures the polysaccharide polymerase CpsH at the division site. Together with the finding that capsule is not produced at the division site in *cpsD* and *cpsC* mutants, these data show that CPS production occurs exclusively at mid-cell and is tightly dependent on CpsD interaction with CpsC. Next, we have analyzed the impact of CpsD phosphorylation on CPS production. We show that dephosphorylation of CpsD induces defective capsule production at the septum together with aberrant cell elongation and nucleoid defects. We observe that the cell division protein FtsZ assembles and localizes properly although cell constriction is impaired. DAPI staining together with localization of the histone-like protein HlpA further show that chromosome replication and/or segregation is defective suggesting that CpsD autophosphorylation interferes with these processes thus resulting in cell constriction defects and cell elongation. We show that CpsD shares structural homology with ParA-like ATPases and that it interacts with the chromosome partitioning protein ParB. Total internal reflection fluorescence microscopy imaging demonstrates that CpsD phosphorylation modulates the mobility of ParB. These data support a model in which phosphorylation of CpsD acts as a signaling system coordinating CPS synthesis with chromosome segregation to ensure that daughter cells are properly wrapped in CPS.

## Author Summary

Bacteria utilize a multi-protein membrane complex to synthesize and export the polysaccharide capsule that conceals and covers the cell. In bacterial pathogens, the capsule protects the cell form opsonophagocytosis and complement-mediated killing. The mechanisms allowing the bacterial cell to maintain this protective capsule during cell growth and division remain unknown. The capsule assembly machinery encompasses a particular type of tyrosine-kinases found only in bacteria, which are called BY-kinases. These kinases are involved in the regulation of several cellular functions including polysaccharide capsule production. Studying the role of BY-kinase represents thus an interesting approach to decipher the mechanisms of capsule synthesis and export. Here, we study the role of the BY-kinase CpsD in the human pathogen *Streptococcus pneumoniae*. We show that CpsD plays a dual function in the pneumococcus. Indeed, CpsD captures the capsule assembly machinery at the site of division, but we also show that CpsD coordinates capsule production with the cell cycle by interacting with the chromosome segregation system. These features provide a simple mechanism to cover the complete surface of the pneumococcal daughter cells. This finding further opens a new view of the function of BY-kinases in the bacterial cell notably in localizing protein complexes in subcellular regions over the cell cycle.

## Introduction

*Streptococcus pneumoniae* is a Gram-positive bacterium usually found as a commensal in healthy adults and children [1]. It does however have the potential to become pathogenic and is a frequent cause of community-acquired diseases. *S*. *pneumoniae* is associated with a variety of infections that can range in severity from otitis media to pneumonia or meningitis [2]. Despite the availability of antibiotics, pneumococcal infections still have high mortality rates and vaccine efficiency drops over time as new and infectious non-vaccine covered serotypes are emerging in clinical isolates [3]. Pneumococcal virulence is strictly dependent on the capsular polysaccharide (CPS) production: non-encapsulated mutants of clinical pneumococcal isolates are non-virulent [4]. The capsule plays a major role in both colonization and persistence of *S*. *pneumoniae* in the infected host due to its ability to form a shield that prevents antibodies and complement components from interacting with their receptors on the host phagocytic cells [5, 6].

**Fig 1:**
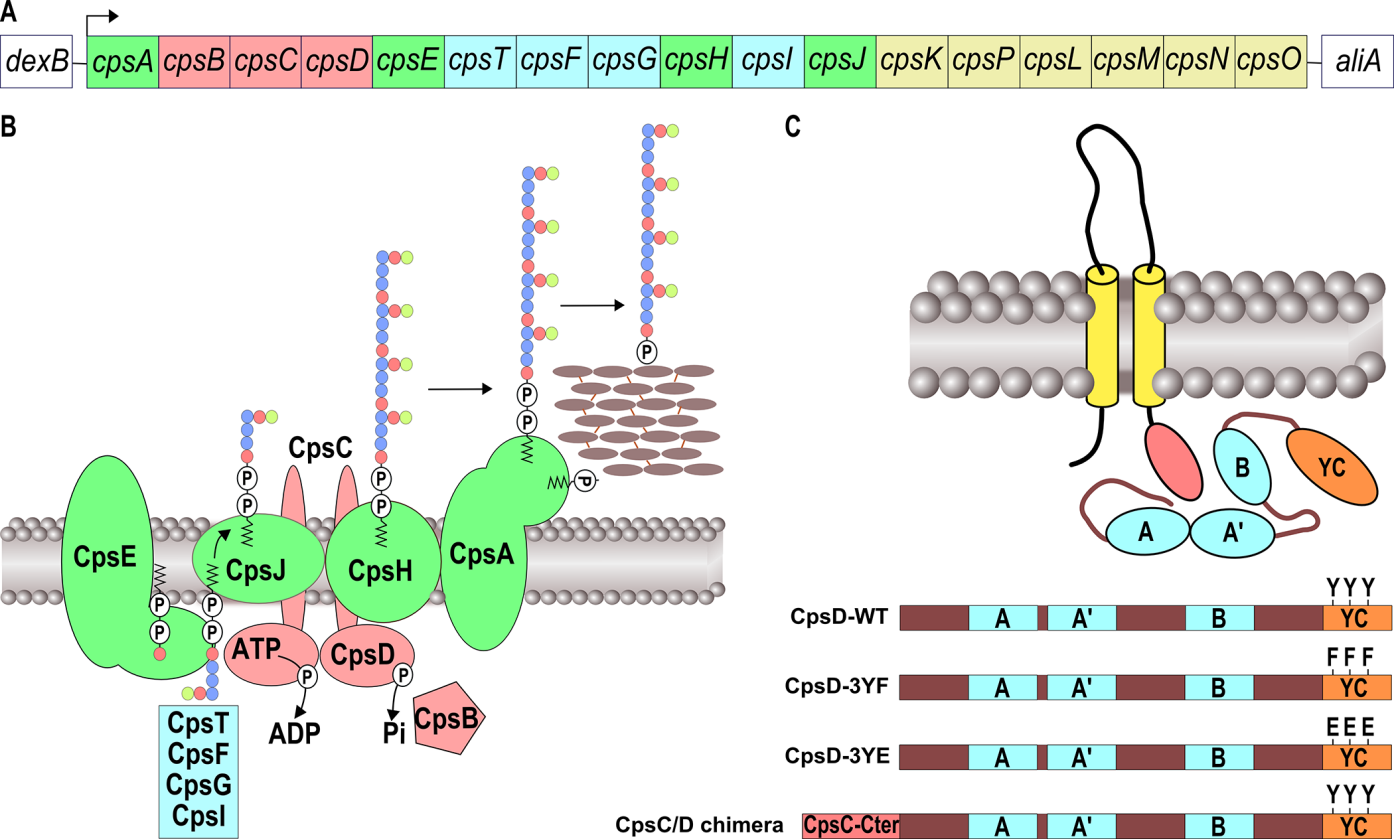
Schematic organization of the pneumococcal CPS machinery and BY-kinase. **(**A) The genetic locus depicts the genes involved in the synthesis and export of the CPS serotype 2 of *S*. *pneumoniae* D39. Genes coding for the CPS assembly machinery, glycosyl-transferases, the CpsBCD phospho-regulatory system and UDP-sugar synthetases are green, blue, red and yellow boxed, respectively. (B) Model of the CPS assembly machinery in the membrane. The color code is the same as in (A). Lipid-carrier-linked CPS subunits are assembled at the inner-side of the membrane by several glycosyl-transferases and then flipped across the membrane in a process requiring the flippase CpsJ. This provides the substrates for CpsH-dependent polymerization. CpsA finally links the polymer to peptidoglycan. (C) Organization of BY-kinases in Firmicutes and features of CpsD mutants. The yellow rods and the red oval show the two transmembrane spanning regions and the cytoplasmic C-terminal end of the membrane modulator required for kinase activation, respectively. The Walker A, A’ and B motifs are indicated in blue. The C-terminal tyrosine cluster encompassing the 3 phosphorylated tyrosines is shown in red. Y substitutions for F or E show phospho-ablative and phospho-mimetic mutations, respectively. To construct the CpsC/D chimera, the cytoplasmic C-terminal end of CpsC (red box) was fused to the N-terminal-end of CpsD.

In all serotypes, the *cps* operon includes serotype-specific genes, encoding enzymes required for the synthesis of specific sugar components, as well as conserved genes encoding proteins essential for capsular synthesis and export (Fig 1A) [7]. Export of the capsule across the plasma membrane occurs by a Wzy-dependent polymerization pathway, analogous to Group 1 CPS biosynthesis in *Escherichia coli* [8, 9] (Fig 1B). The 5’ region of the locus encodes the *cpsA*, *cpsB*, *cpsC* and *cpsD* genes, (also known as *wzg*, *wzh*, *wzd* and *wze*) (Fig 1A). CpsA was shown to interact with the pyrophosphoryl-lipid carrier of the polysaccharide precursor and is proposed to attach capsular polysaccharide to cell wall peptidoglycan [10]. *cpsB*, *cpsC and cpsD* constitute a phosphoregulatory system that controls the polysaccharide assembly machinery encompassing a glycosyl-transferase (CpsE), a flippase (CpsJ) and a polymerase (CpsH) (Fig 1B) [9]. CpsB is a metal-dependent phosphotyrosine-protein phosphatase of the PHP family [11] whereas CpsC and CpsD constitute a so-called BY-kinase, a particular type of tyrosine-autokinase, which shares no resemblance with eukaryotic tyrosine-kinase and is conserved among most bacterial phyla [12–14].

BY-kinases consist of two main structural domains: an N-terminal extracellular domain flanked by two transmembrane helices and a cytoplasmic C-terminal domain, harboring the kinase activity [15]. In Firmicutes, these domains are encoded by two successive genes, and are therefore present as separate polypeptide chains, one cytoplasmic and the other in the membrane (Fig 1C). The two polypeptides need to interact to form an active BY-kinase [16]. The crystal structure of the BY-kinase CapB from *Staphylococcus aureus* showed that the cytoplasmic C-terminal end of the transmembrane modulator CapA is required for the activation of the cytoplasmic kinase CapB [17]. More precisely, the C-terminal extremity of CapA forms a αA-ßA motif complementing the catalytic site of CapB and stabilizing the ATP molecule. The cytoplasmic domain of BY-kinases is able to autophosphorylate on several tyrosines forming a C-terminal tyrosine cluster motif (Fig 1C) [18, 19]. Although the detailed mechanisms by which BY-kinases promote CPS synthesis and export remain elusive, it has been proposed that cycling between phosphorylated and non-phosphorylated forms of the BY-kinase, regulated by the cognate phosphotyrosine-phosphatase, is required for proper synthesis and export of the polysaccharide polymer [20–23].

The single BY-kinase produced by most of the 93 *S*. *pneumoniae* serotypes [9] comprises the transmembrane modulator CpsC and the cytoplasmic kinase domain CpsD [12, 13]. Several studies reported that autophosphorylation of CpsD in the tyrosine cluster negatively regulates CPS production [18, 24]. Moreover, evidence was provided that CpsD tyrosine-kinase activity influences capsule production and modulates invasive pneumococcal disease [12, 25]. Interestingly, it was recently shown that CpsD and CpsC both localize at the division site in the serotype 14 strain ATCC6314 [13]. In addition, capsule is absent from the division site and detected only at the old cell halves in the absence of either CpsD or CpsC. Collectively, it is hypothesized that the CPS assembly machinery would adopt two distinct localizations: one around the cell and one at the division site captured by CpsC and CpsD for septal CPS production. In the latter, CpsC and CpsD would either act as activators of CPS export or function as the exporter [13].

Here, we have investigated the role of CpsD activation by CpsC as well as the impact of CpsD autophosphorylation on capsule production in the well-studied serotype 2 strain D39 [26]. We first show that the C-terminal cytoplasmic end of CpsC is required for CpsD autophosphorylation and localization at the division septum. Analysis of CPS production together with imaging of the polysaccharide polymerase CpsH localization demonstrates that CPS are exclusively produced at the division septum in WT cells and challenges the two-machine model for CPS assembly. Strikingly, we also observe that cells producing a non-phosphorylatable variant of CpsD display defective capsule production at the septum together with aberrant elongated shape with multiple non-constricted septa, nucleoid defects and reduced dynamics of the chromosome segregation protein ParB. In line with a role of CpsC and CpsD in controlling the pneumococcal cell cycle, it was shown that BY-kinases have homology to the large P-loop NTPase superfamily [27] that includes ParA-like proteins, which are involved in chromosome segregation by interacting with ParB [28]. Molecular modeling confirms that CpsD displays structural similarities with ParA. Interestingly, we found that CpsD interacts with ParB *in vitro* and that the stability of the CpsD/ParB complex is modulated by CpsD phosphorylation *in vivo*. These observations show that CPS production is tightly connected with the cell cycle and support a model wherein crosstalk between CpsD and ParB, modulated by CpsD autophosphorylation, signals the status of CPS production to the proteins in charge of chromosome segregation, thus ensuring coordination between encapsulation and cell division.

## Results

### The cytoplasmic C-terminal end of CpsC is required for CpsD phosphorylation and localization at the division site

**Fig 2:**
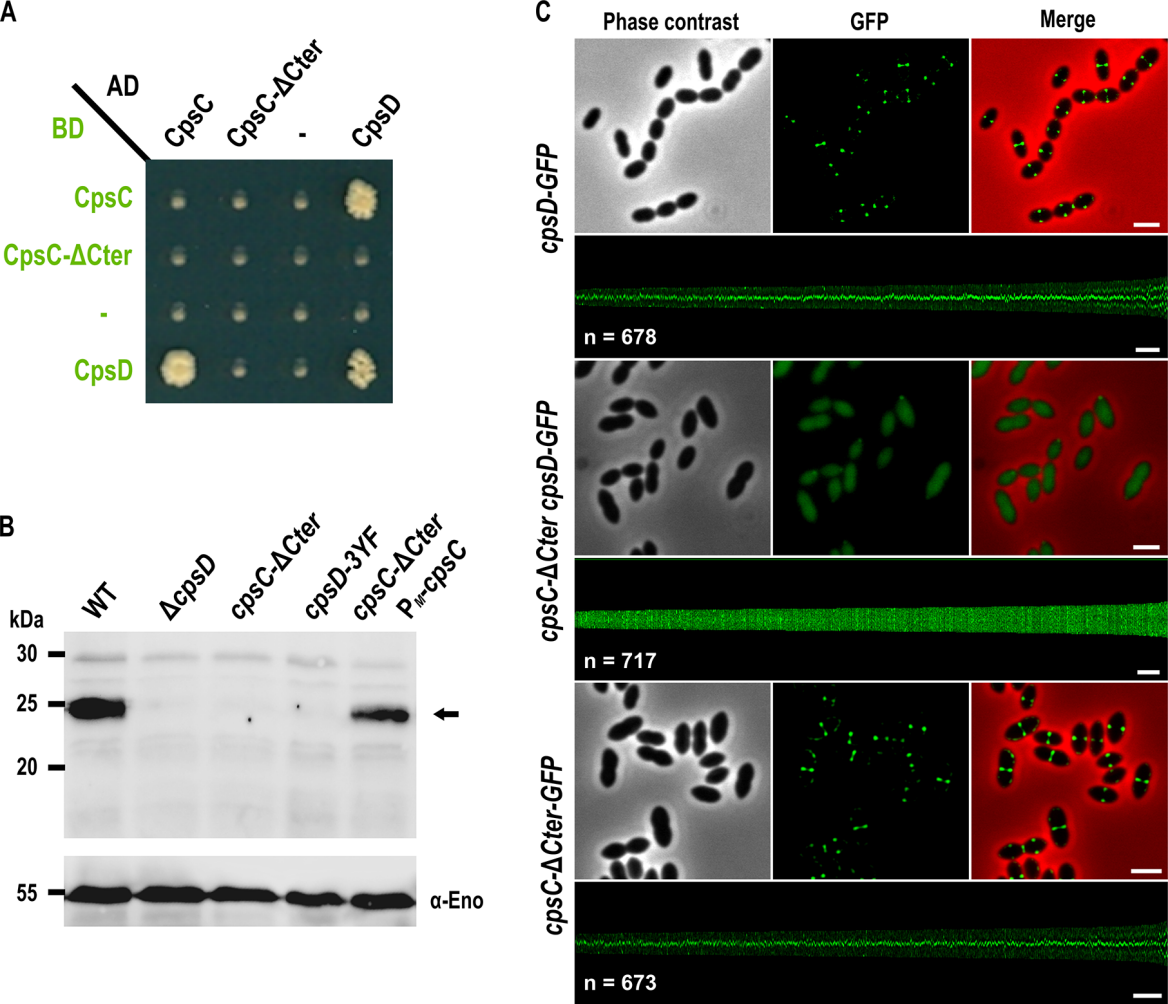
Role of the cytoplasmic C-terminal end of CpsC in CpsD phosphorylation and localization. (A) Protein-protein interaction between CpsD, CpsC, and CpsC-ΔCter assayed by yeast two-hybrid. AD and BD refer to activating and DNA binding domain of Gal4 fused to CpsC, CpsC-ΔCter, and CpsD. (B) *in vivo* phosphorylation of CpsD in WT, Δ*cpsD*, *cpsC-*Δ*Cter*, *cpsD-3YF* and *cpsC-*Δ*Cter* P_*M*_-*cpsC* cells. The CpsD (calculated molecular mass 24,83 kDa) phosphorylation signal is indicated by an arrow. The Western immunoblot was probed with anti-phosphotyrosine antibodies. To estimate the relative quantity of proteins in crude extract and to compare the different lanes, we used the enolase as an internal standard. The enolase was detected using specific antibodies (α-Eno) as described in [32] and is presented in the lower part of the figure (C) Localization of CpsD-GFP in WT (upper row) and *cpsC-*Δ *Cter* (middle row) cells and *CpsC-*Δ*Cter-GFP* in WT cells (lower row). Phase contrast (left), GFP fluorescent signal (middle) and overlays (right) between phase contrast (red) and GFP (green) images are shown. The map of fluorescence profiles of WT and *cpsC-*Δ*Cter* cells sorted according to their length is presented. The total integrated fluorescence of each cell is plotted as function of its cell length (y-axes) and all cells are plotted with increasing cell length from left to right (x-axes). n indicates the number of cells analyzed in each panel. Scale bar, 2 μm.

Structural studies of BY-kinases have established that the C-terminal peptide of the transmembrane modulator specifically interacts and activates the cytoplasmic catalytic domain [17]. We first tested whether the C-terminal end of CpsC (CpsC-Cter) is required for CpsD autophosphorylation as it is the case for BY-kinases from other Firmicutes [16, 29, 30]. To do that, we first analyzed by yeast two-hybrid assays the ability of a derivative of CpsC lacking the C-terminal 30 amino acids (CpsC-ΔCter) to interact with CpsD. We observed that full-length CpsC interacts efficiently with CpsD while this interaction was abolished in the absence of CpsC-Cter (Fig 2A). As expected for a BY-kinase [17], CpsD interacted with itself. Next, we constructed a nonpolar markerless mutant strain expressing CpsC-ΔCter (*cpsC-*Δ*Cter* strain) and analyzed CpsD autophosphorylation using anti-phosphotyrosine antibodies (Fig 2B). Non-polarity of the deletion of *cpsC-Cter* was confirmed by analyzing the expression of CpsD and CpsH fused to GFP (see below) in WT and *cpsC-*Δ*Cter* strains (S1A and S2 Figs) and partial restoration of capsule production in the *cpsC-*Δ*Cter* mutant carrying a copy of *cpsC* at the ectopic *amiF/treR* locus under the control of the maltose inducible promoter P_*M*_ [31] (S1B and S1C Fig). No phosphorylation signal was detected for CpsD in the *cpsC-*Δ*Cter* mutant (Fig 2B). As controls, CpsD was efficiently phosphorylated in the wild-type strain whereas no phosphorylation signal was detected in a mutant deficient for CpsD *(*Δ*cpsD*) or a strain producing CpsD mutated on the three tyrosines of its C-terminal tyrosine cluster motif. In addition, CpsD phosphorylation was partially restored in the *cpsC*-Δ*Cter* strain complemented with P_*M*_-*cpsC* (Fig 2B). The partial restoration of CpsD phosphorylation and capsule synthesis observed for the *cpsC-*Δ*Cter* P_*M*_ *-cpsC* strain suggests that production of CpsC-ΔCter interferes with the ability of native CpsC to interact with CpsD and/or to function in the CPS assembly machinery. Next, we analyzed the effect of the CpsC C-terminal deletion (CpsC-ΔCter) on CpsD localization. For this purpose, we constructed a C-terminal monomeric GFP fusion to CpsD. The CpsD-GFP fusion is stable and functional since cells grew normally and displayed normal CPS production patterns (S3 and S4 Figs) [24]. As previously shown in a serotype 14 strain [13], CpsD-GFP also localized at midcell in serotype 2 D39 cells (Fig 2C). However, CpsD delocalized to the cytoplasm in *cpsC-*Δ*Cter* cells whereas CpsC-ΔCter-GFP still localized at midcell (Fig 2C). Altogether, these observations show that the C-terminal end of CpsC is required to position CpsD at the division site and trigger its autophosphorylation.

### The cytoplasmic C-terminal end of CpsC is required for septal localization of the polysaccharide polymerase CpsH and CPS production

**Fig 3:**
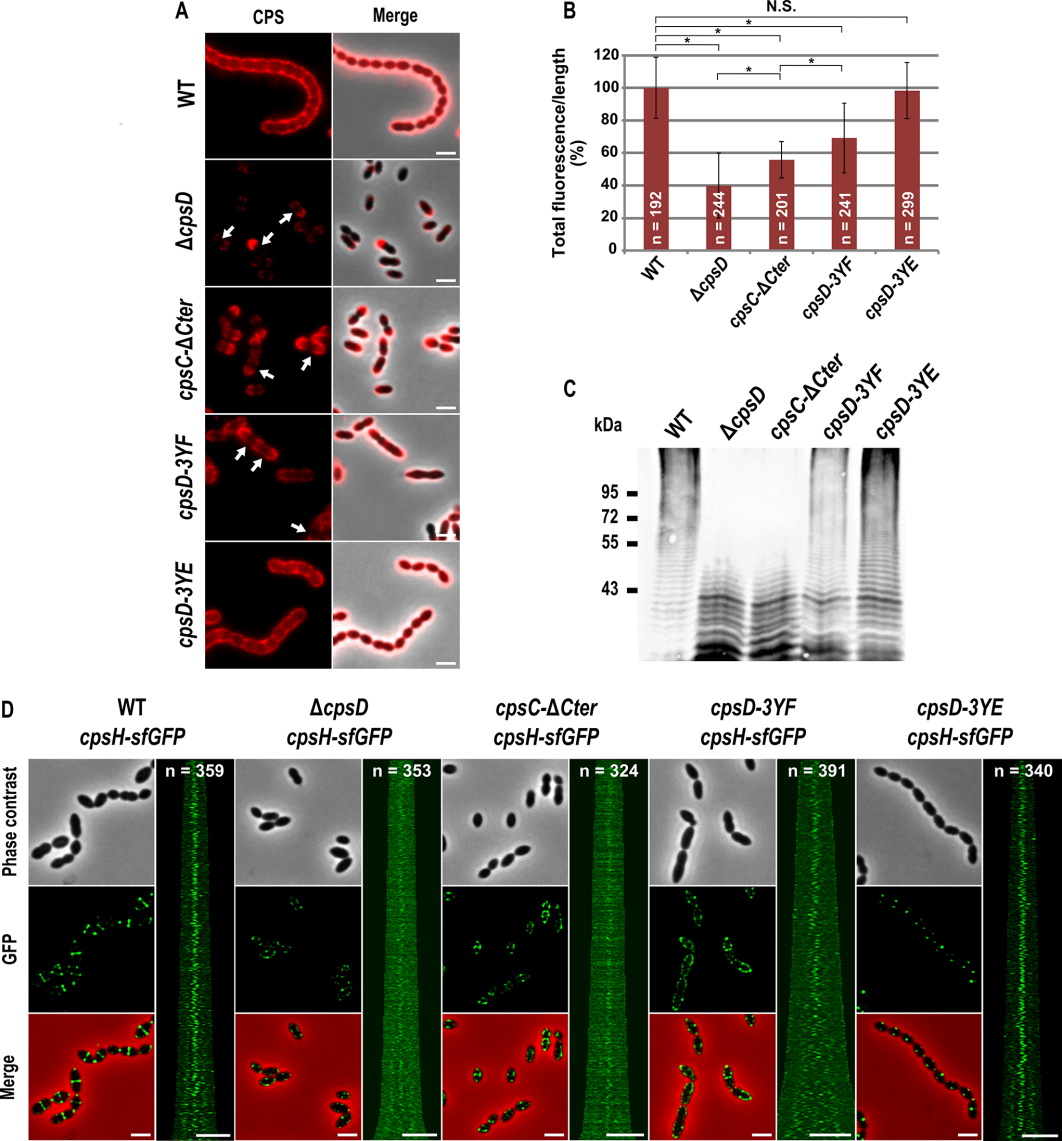
Expression of CPS and localization of CpsH. (A) Detection of CPS in living WT and mutants cells. CPS were immunodetected with an rabbit anti-serotype 2 CPS polyclonal antibody. CPS fluorescent signal (red, left row) and overlays between phase contrast and CPS fluorescence images (right row) are shown. Arrows indicate the absence of CPS at the division site. (B) Quantification of the CPS fluorescent signal in living WT and mutant strains observed in (A). The relevance of the quantification was confirmed statistically (Mann-Whitney rank sum test). Asterisks indicates p-value<0.0001 while NS means that the p-value is >0.05. n indicates the number of cells analyzed and standard deviation from the fluorescence of the n cells is indicated with error bars. (C) Detection of cell-associated CPS in the WT strain and mutants. The same amount of samples from culture grown until OD_550_ = 0.3 was loaded in each lane (see Materials and Method). The immunoblot was probed with a rabbit anti-serotype 2 CPS polyclonal antibody. (D) Localization of CpsH-sfGFP produced in WT strain and mutants. From left to right: WT, Δ*cpsD*, *cpsC-*Δ*Cter*, *cpsD-3YE* and *cpsD-3YF* cells. Each panel consists of a phase-contrast image, a GFP fluorescent image, an overlay between phase contrast and GFP images and a map of CpsH-sfGFP fluorescence profiles of 350–400 cells. Scale bar, 2 μm.

Our observations prompted us to analyze CPS synthesis in the *cpsC-*Δ*Cter* mutant. For that, we used anti-serotype 2 capsule antibodies and localized CPS by immunofluorescence microscopy. Consistent with observations reported by Henriques and co-workers [13], CPS were not produced at the division site but only at the old cell halves in the Δ*cpsD* mutant while CPS were detected over the entire surface of wild-type cells (Fig 3A). We also observed that CPS were absent from the division septa in the *cpsC-*Δ*Cter* mutant (Fig 3A). These data show that the absence of CpsC C-terminus, and consequently CpsD phosphorylation and localization at midcell, alters the localization and/or the activity of the capsule assembly machinery. To test these hypotheses, we quantified the CPS fluorescence signal in living cells and immunodetected the total fraction of CPS produced by our mutants using anti-type 2 capsule antibodies. We observed a striking CPS production and polymerization defect in both *cpsC-*Δ*Cter* and Δ*cpsD* mutants compared to the WT strain (Fig 3B and 3C). This defect led to the accumulation of low molecular weight polysaccharides (Fig 3C). Next, we used the polymerase CpsH as a marker to localize the capsule assembly machinery. CpsH is a predicted membrane protein with both N- and C-terminal ends located outside the cell [9]. It was shown that superfolder GFP (sfGFP), in certain fusions, can fluoresce in the extracellular milieu [33]. We thus constructed a strain expressing CpsH fused to sfGFP at its C-terminus. WT cells producing CpsH-sfGFP as the only source of CpsH from their endogenous chromosomal locus grew and produced capsule similarly to WT cells attesting that the fusion CpsH-sfGFP is functional (S3 Fig). As shown in Fig 3D, we found that CpsH-sfGFP localized exclusively at the division septum in WT cells. Interestingly, CpsH partially delocalized in both *cpsC-*Δ*Cter* and Δ*cpsD* mutants, and the signals were not exclusively present at the division septum (Fig 3D). CpsH-sfGFP was stable and produced at similar amount in WT and *cpsC-*Δ*Cter* and Δ*cpsD* mutants (S2 Fig). These data suggest that the capsule assembly machinery localizes at the division site to assemble CPS. In addition, the localization and the activity of the capsule assembly machinery are both dependent on CpsD localization at the division site.

### Non-phosphorylated CpsD hinders capsule production and cell division

**Fig 4:**
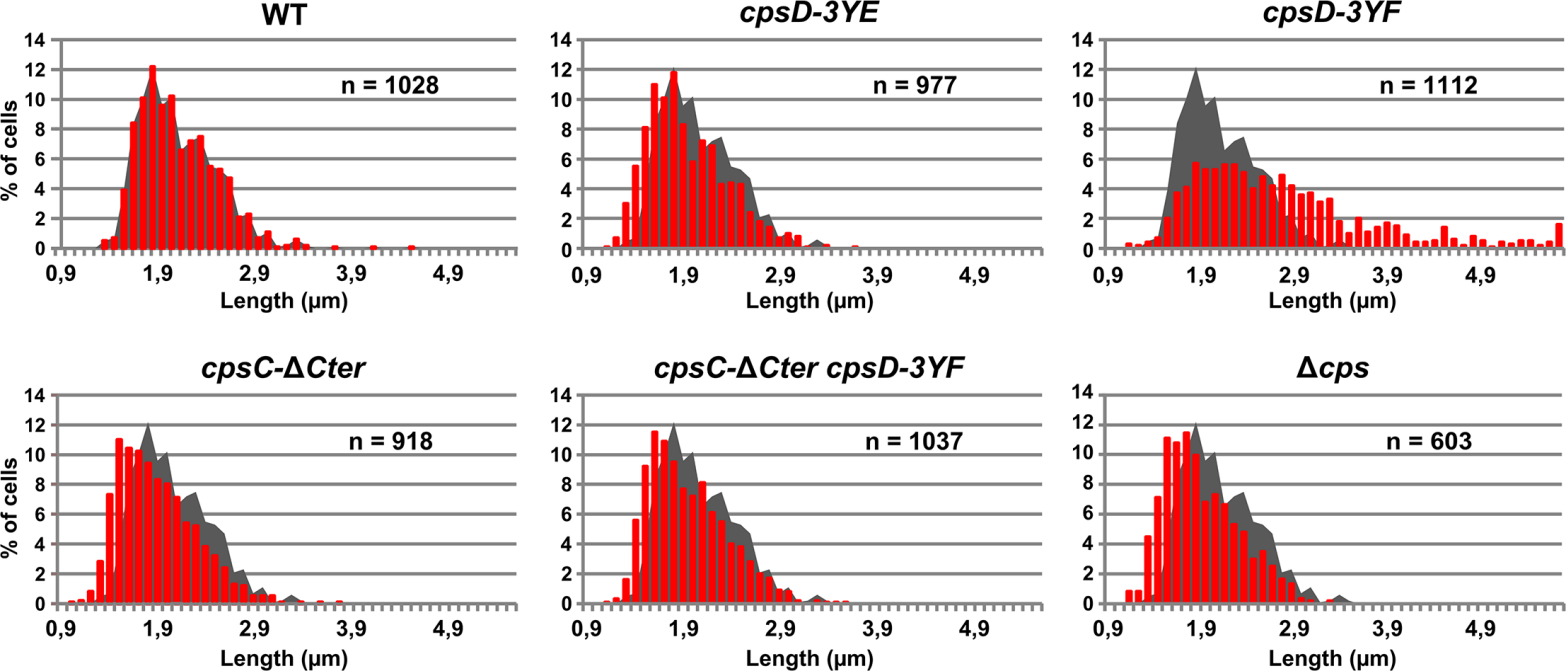
Cell length distributions of pneumococcal WT strain and mutants. Cell length distribution of WT, *cpsD-3YE*, *cpsD-3YF*, *cpsC-*Δ*Cter*, *cpsC-*Δ*Cter cpsD-3YF* and Δ*cps* strains. In each diagram, the grey shadow shows the distribution of WT cells for comparison. n indicates the number of cells analyzed.

To better understand the role of CpsD phosphorylation at the division site in CPS production, we constructed two mutant strains expressing either non-phosphorylated CpsD (*cpsD-3YF*) or its phosphomimetic form (*cpsD-3YE*). For that, each of the 3 tyrosines of the tyrosine cluster of CpsD was substituted either for phenylalanine or glutamic acid. Then, we analyzed these strains for capsule production and CpsH localization. As shown in Fig 3A and 3B, *cpsD-3YE* cells displayed CPS localization pattern and quantification indistinguishable from that of WT cells even if the capsular halo around WT cells suggested that CPS could be in less tighter association with the cells than in *cpsD-3YE* cells. CpsH-sfGFP was also found to localize properly at the septum in *cpsD-3YE* cells (Fig 3D). However, by measuring cell lengths, *cpsD-3YE* cells were significantly shorter (1.88 μm +/- 0.33) than WT cells (2.02 μm +/- 0.32, p<0.0001, Mann Whitney rank sum test) (Fig 4). Furthermore, *cpsD-3YF* cells displayed capsule mainly at the old cell poles as observed in Δ*cpsD* cells (Fig 3A). Strikingly, 26.6% of *cpsD-3YF* cells possessed an elongated cell shape (> 3 μm). This aberrant cell length of > 3 μm was observed in less than 1.4% of WT or *cpsD-3YE* cells (Fig 4). Furthermore, CpsH-sfGFP partially delocalized and formed several foci around the cell in the *cpsD-3YF* mutant although CpsH-sfGFP was stable and produced at similar amount in WT and *cpsD-3YF* and *cpsD-3YE* mutants (S2 Fig). In agreement with these observations, quantification of CPS fluorescence signal and western immunoblotting using anti-serotype 2 capsule antibodies showed that CPS production was hampered in the *cpsD-3YF* mutant compared to that of WT and *cpsD-3YE* cells (Fig 3B and 3C). However, CPS polymerization remained clearly more effective than in *cpsC-*Δ*Cter* and Δ*cpsD* mutants suggesting that beyond CpsD phosphorylation, deletion of *cpsD* or *cpsC-Cter* further alters the global functioning of the CPS assembly machinery (Fig 3B and 3C). These data show that permanent dephosphorylation of CpsD is detrimental for localizing the capsule synthesis machinery at the division site and causes problems with cell division, thus suggesting a link between defective capsule synthesis and aberrant elongation of cells.

### Septal localization of non-phosphorylated CpsD impairs cell division

**Fig 5:**
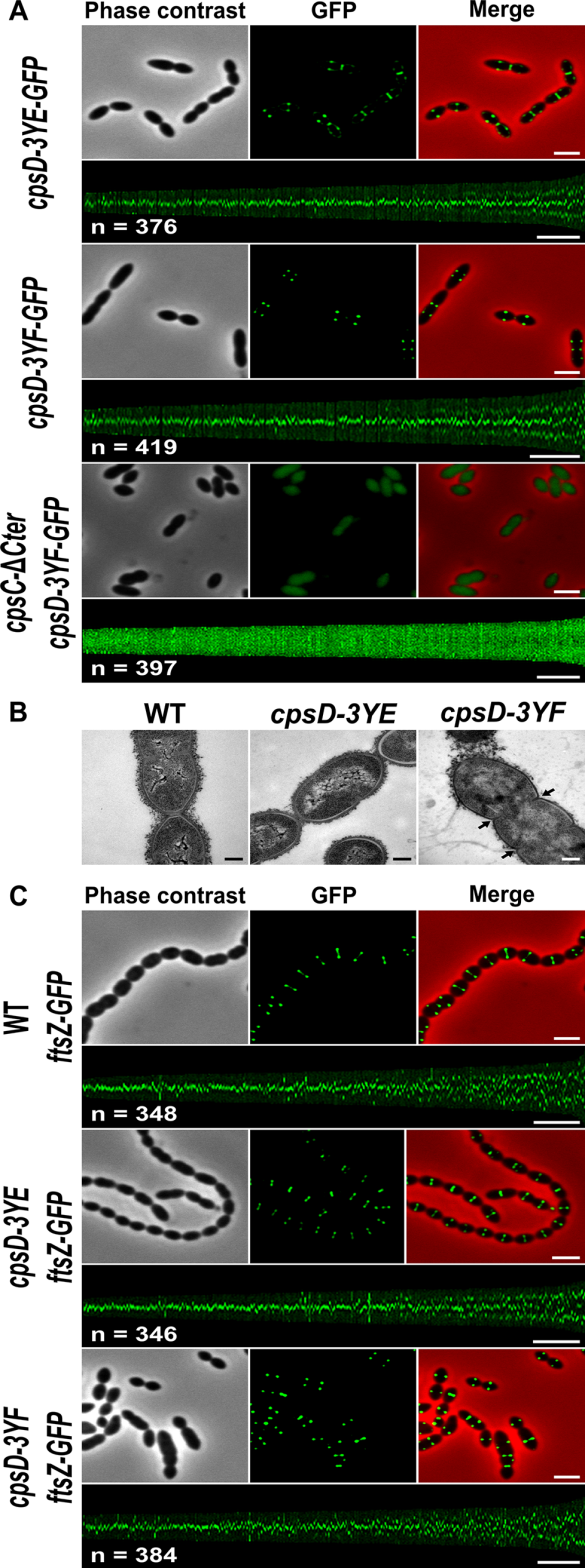
Analysis of *cpsD-3YF* and *cpsD-3YE* mutants. (A) Localization of GFP fused to CpsD-3YE in WT cells (upper row) and CpsD-3YF in WT (middle row) and *cpsC-*Δ*Cter* (lower row) cells. Phase contrast (left), GFP fluorescent signal (middle) and overlays (right) between phase contrast (red) and GFP (green) images are shown. The map of CpsD-3YE-GFP and CpsD-3YF-GFP fluorescence profiles of 350–400 cells is shown. Scale bar, 2 μm. (B) Transmission electron micrograph of WT (left panel), *cpsD-3YE* (middle panel) and *cpsD-3YF* (right panel) strains. Arrows indicate defective septal initiations. Scale bar, 0.2 μm. (C) Localization of FtsZ-GFP in WT, *cpsD-3YE* and *cpsD-3YF* cells. Phase contrast (left), GFP fluorescent signal (middle) and overlays (right) between phase contrast (red) and GFP (green) images as well as the map of FtsZ-GFP fluorescence profiles of 350–400 *cpsD-3YF* and *cpsD-3YE* cells are shown. Scale bar, 2 μm.

We hypothesized that the absence of CpsD phosphorylation could be detrimental for its localization at midcell. To test whether the phosphorylation state of CpsD influences its localization, we constructed strains producing C-terminal GFP fusions to either CpsD-3YE (*cpsD-3YE-GFP*) or CpsD-3YF (*cpsD-3YF-GFP*). Both types of tyrosine substitutions did not affect the stability and the production of the CpsD-GFP fusion (S4 Fig). Fluorescence microscopy indicated that these mutations do not affect septal localization (Fig 5A). Therefore, we questioned why expression of *cpsD-3YF* induces cell elongation whereas cells expressing CpsC-ΔCter, in which CpsD autophosphorylation is prevented (Fig 2B), are not elongated (Figs 2B and 4). To reconcile this apparent contradiction, we constructed mutant strains expressing *cpsC-*Δ*Cter* and *cpsD-3YF* fused or not to *gfp* (*cpsC-*Δ*Cter-cpsD-3YF-gfp* and *cpsC-*Δ*Cter-cpsD-3YF*). Consistent with our observations presented in Fig 2C, CpsD-3YF-GFP delocalized in the cytoplasm in the absence of the cytoplasmic C-terminal end of CpsC (Fig 5A). However, *cpsC-*Δ*Cter-cpsD-3YF* cells did not display an aberrant elongated cell shape phenotype anymore indicating that the deletion of CpsC-Cter suppresses cell elongation of the *cpsD-3YF* mutant (Fig 4). Together, these data suggest that the presence of non-phosphorylated CpsD at the division site partially inhibits cell division, leading to an elongated cell shape phenotype.

### *cpsD-3YF* cells display defective cell constriction and aberrant nucleoid morphology

To better analyze the ultrastructure of elongated *cpsD-3YF* cells, we examined these cells by transmission-electron microscopy (TEM). As controls, WT and *cpsD-3YE* cells were found to possess the characteristic ovoid cell shape (Figs 5B and S5). By contrast, examination of *cpsD-3YF* mutant confirmed the severe disturbed cell morphology observed by phase-contrast microscopy (Fig 5B). In addition, these images showed the presence of multiple septal initiations on each side of the long cell axis, indicative of impaired cell constriction (Fig 5B). In addition, and in agreement with CPS immunolabelling (Fig 3A), CPS were primarily detected at the pole in *cpsD-3YF* cells observed by electron microscopy (Fig 5B).

To further investigate the division defects of the *cpsD-3YF* mutant strain, we analyzed the localization of the major cell division protein FtsZ that forms contractile rings at mid-cell [34]. As expected, FtsZ-GFP [35] localized at mid-cell in WT cells as well as in *cpsD-3YE* cells (Fig 5C). In elongated *cpsD-3YF* cells, FtsZ-GFP fluorescence showed a ladder localization pattern indicating that it is properly recruited to each non-constricted septum (Fig 5C). This observation shows that the division defects of *cpsD-3YF* cells are not due to altered Z-ring assembly at the division site.

**Fig 6:**
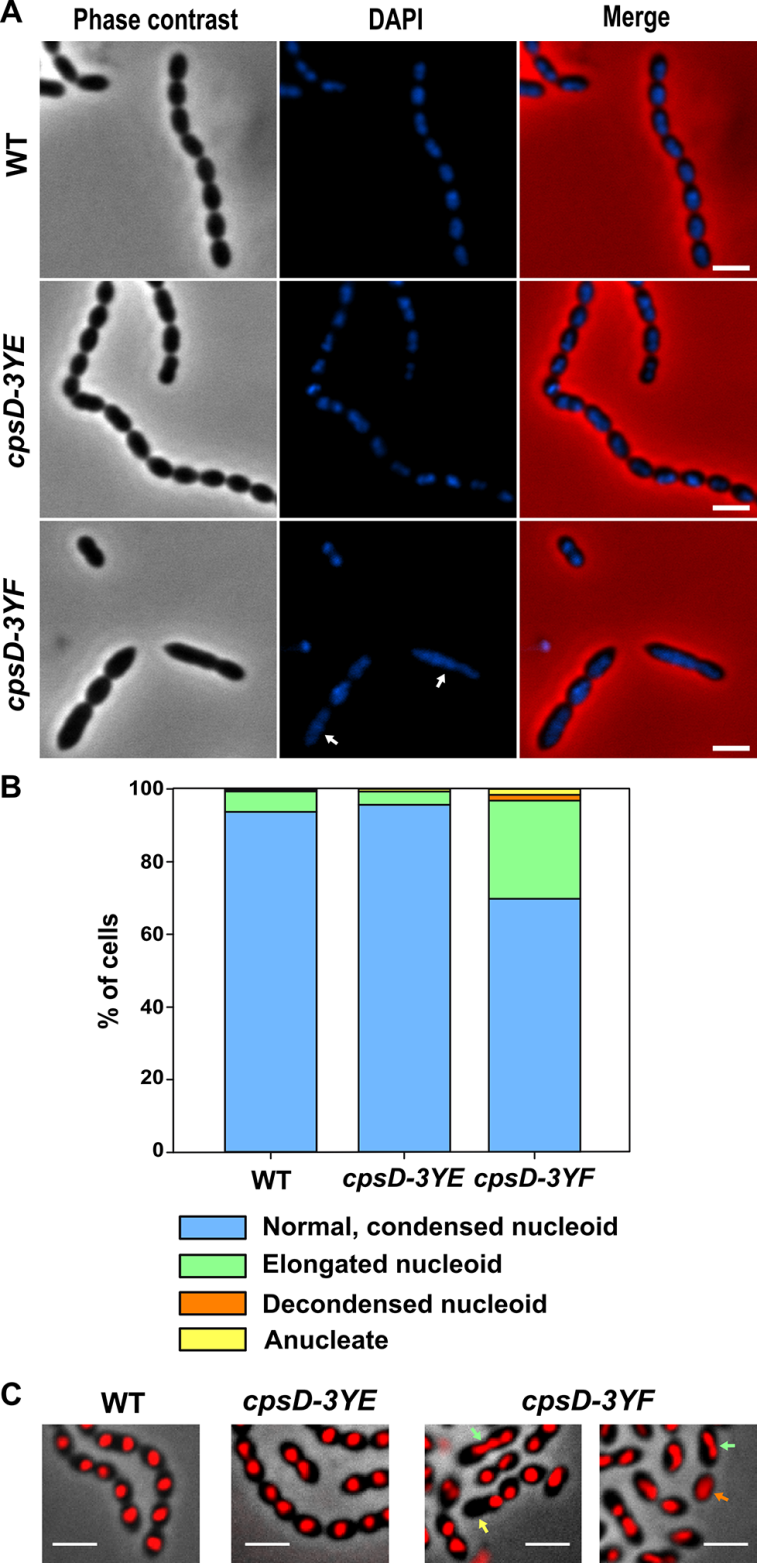
Visualization of nucleoids in WT and *cpsD* mutant strains. (A) Localization of nucleoids was analyzed using DAPI staining in WT (upper row), *cpsD-3YE* (middle row) and *cpsD-3YF* (lower row) cells. From left to right: Phase contrast, DAPI fluorescent signal and overlays between phase contrast and DAPI images. Scale bar, 2 μm. (B) Nucleoid shape of WT, *cpsD-3YE* and *cpsD-3YF* live cells expressing *hlpA-rfp* as marker for the nucleoids. The cells were split in categories based on manual visual inspection of their nucleoids; normal, condensed nucleoids, elongated nucleoids, decondensed nucleoids or anucleate cells, and the graph shows the percentage of cells in each category. For each strain, more than 400 cells were analyzed from images obtained during time-lapse imaging. (C) Microscopy images of WT, *cpsD-3YE* and *cpsD-3YF* cells expressing *hlpA-rfp*. An overlay of phase contrast images and RFP images obtained during time-lapse imaging are shown. Examples of elongated nucleoids (green arrow), decondensed nucleoids (orange arrow) and anucleate cells (yellow arrow) are indicated. Scale bar, 2 μm.

*S*. *pneumoniae* does not contain homologs of proteins involved in nucleoid occlusion, and it was shown that the cell division machinery assembles over the nucleoid and that septation occurs just after chromosomes splitting, indicating that DNA itself can act as a physical barrier for cell division [36]. In line with this idea, mutants affected in chromosome segregation are often elongated [36, 37]. To test whether the non-phosphorylated CpsD mutant induces chromosome segregation defects that could lead to the elongated cell phenotype, we analyzed nucleoid morphology using DAPI staining. As shown in Fig 6A, the nucleoid is properly condensed and segregated in WT and *cpsD-3YE* cells. However, DAPI staining revealed abnormal elongated nucleoids in *cpsD-3YF* cells (Fig 6A). To quantify these nucleoid defects, we used the histone-like HlpA-RFP fusion as a chromosome marker in live cells [36]. As expected, 93.7% of WT cells displayed normal, well-condensed nucleoid(s) (Fig 6B and 6C). The same observation (95.7% normal nucleoids) was made in the *cpsD-3YE* mutant confirming that the expression of CpsD-3YE as the sole source of CpsD does not impact the cell cycle. The situation differed in *cpsD-3YF* cells in which only 69.8% of cells displayed well condensed nucleoid(s). In addition, 3.2% of *cpsD-3YF* cells were either devoid of nucleoid or presented an uncondensed nucleoid (Fig 6B and 6C). These defects are significantly more abundant than in WT and *cpsD-3YE* cells in which they are observed in only 0.6% and 0.7% of cells, respectively. More importantly, we found that nucleoids are elongated and abnormally shaped in 27.0% of *cpsD-3YF* cells compared to 5.7% and 3.6% of WT and *cpsD-3YE* cells, respectively (Fig 6B and 6C). Altogether, these observations show that chromosome replication and/or segregation is impaired in the *cpsD-3YF* mutant, suggesting that expression of non-phosphorylated CpsD at the septum could interfere with these processes.

### ParB mobility is modulated by CpsD phosphorylation

**Fig 7:**
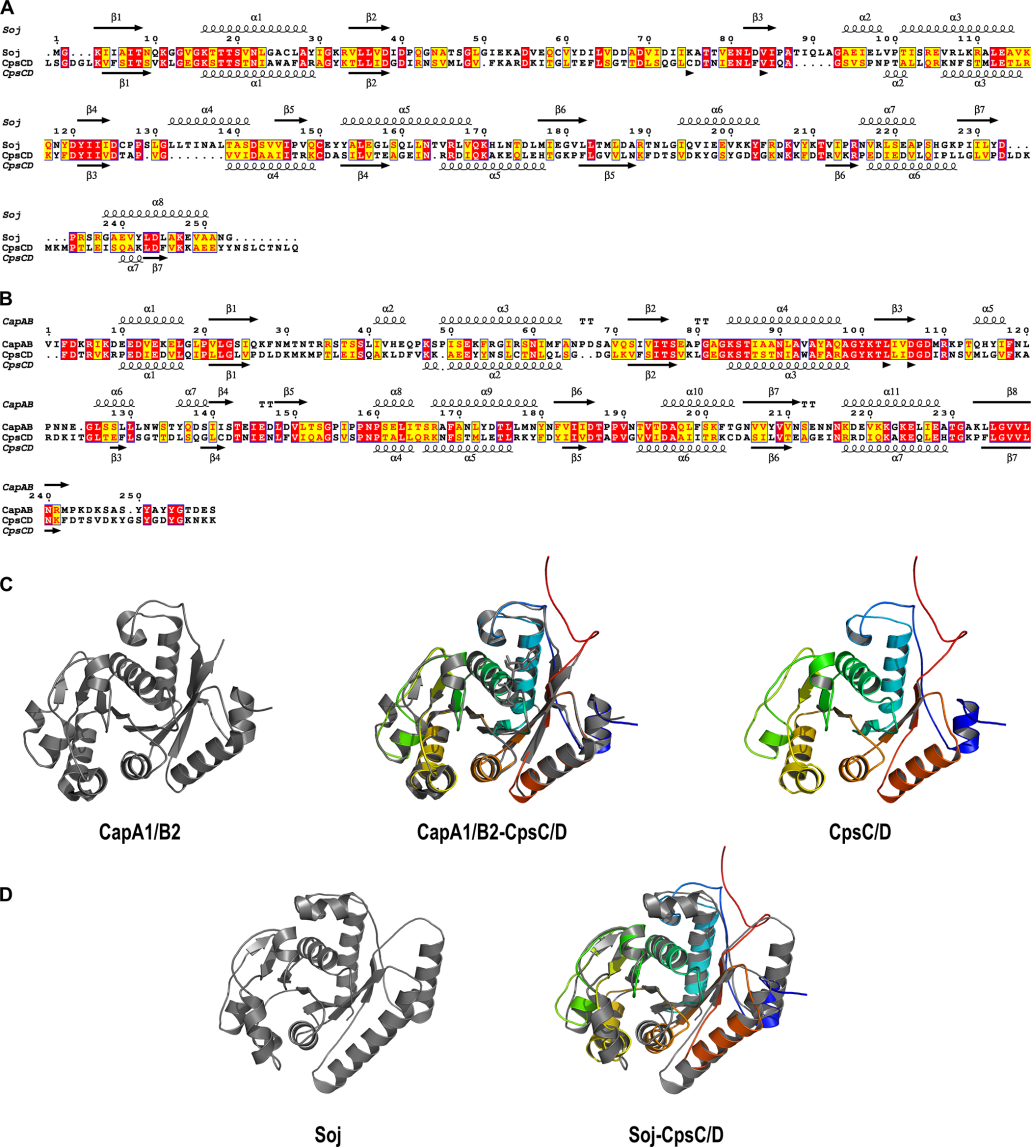
Sequences alignments, secondary structure predictions and molecular modeling of the CpsC/D chimera. Comparison of the amino-acid sequences and secondary structures of (A) CpsC/D with Soj from *Bacillus subtilis* and (B) CpsC/D with the BY-kinase CapA1/B2 from *S*. *aureus*. ß and α indicate ß-sheets and α-helices, respectively. Identical and similar amino-acids are red and yellow boxed, respectively. (C) Modeled 3D-structure of CpsC/D (right panel) using the calculated structure of the BY-kinase CapA1/B2 (PDB 3BFV) (left panel). The merge structures are presented in the middle panel. (D) Superimposition (right panel) of the CpsC/D structural model shown in (C) with the calculated structure of Soj (PDB 2BEK) (left panel) from *Thermus thermophilus*. The proteins are shown as cartoon.

**Fig 8:**
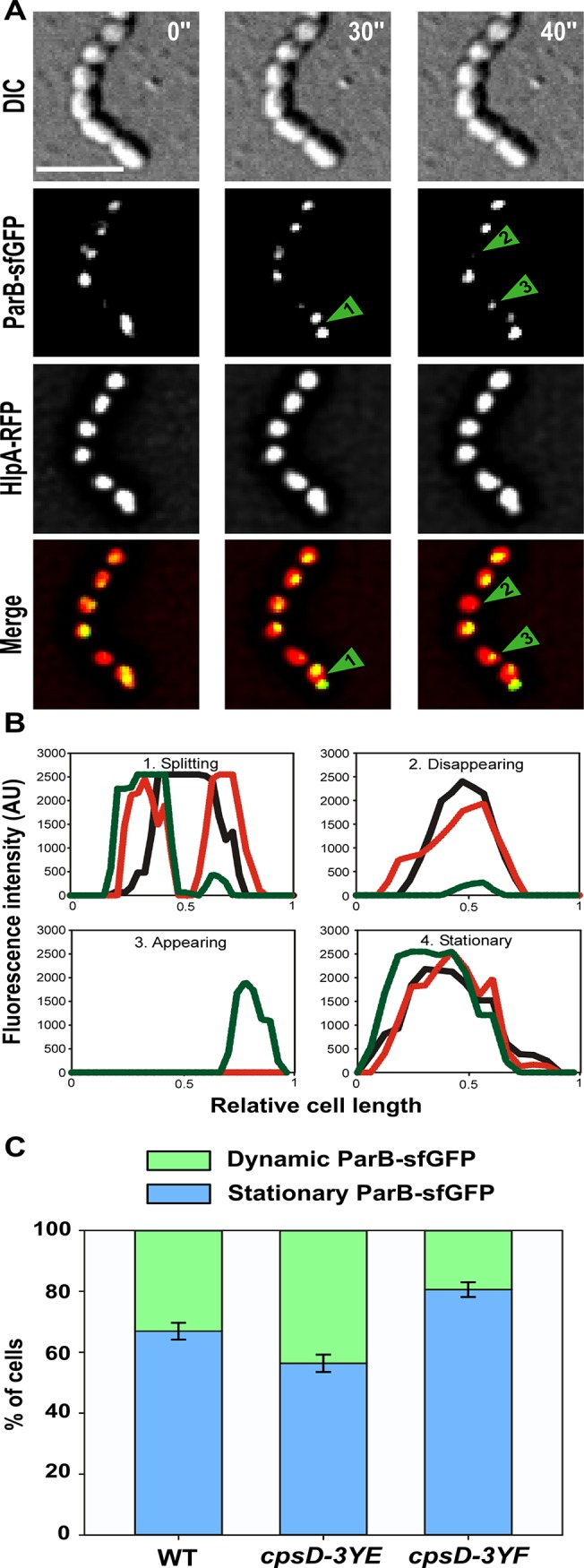
ParB dynamics in *cpsD* mutants. (A) TIRF microscopy time-lapse imaging of WT cells to show dynamics of ParB-sfGFP. Phase contrast, GFP and RFP images were taken every 10 sec for 40 sec. Examples of ParB-sfGFP foci which are dynamic (splitting (1), disappearing (2), appearing (3)) or stationary (4) during this short time-lapse are indicated with arrows. (B) Plot of intensity values from TIRF time-lapse imaging of four cells from (A). The *x*-axis corresponds to the relative length of the cell, while the *y*-axis indicates the arbitrary intensity value for the GFP channel. Intensity plots after 0, 30 and 40 seconds of the time-lapse are shown in black, red and green, respectively. Cells with examples of dynamic ParB-sfGFP signals (splitting, disappearing or appearing) and stationary ParB-sfGFP signal are shown. (C) Quantification of the dynamics of ParB-sfGFP in WT, *cpsD-3YE* and *cpsD-3YF* cells by TIRF time-lapse imaging. The ParB-sfGFP foci in cells were categorized as either stationary or dynamic (*i*.*e*. ParB-sfGFP foci splitting, appearing or disappearing during the time-lapse) as shown in panel A and B. For each strain, the dynamics of ParB-sfGFP was followed in more than 300 cells from three separate experiments. Standard errors are shown.

To test whether initiation of DNA-replication is perturbed when CpsD is not phosphorylated, we performed marker frequency analysis using quantitative real-time PCR [38]. Determining the origin-to-terminus ratios showed no significant difference between WT and *cpsD-3YF* cells (S6 Fig). Interestingly, the chimera CpsC/D composed by CpsD fused to the C-terminal end of CpsC exhibits 20% identity and 52.4% similarity to the *Bacillus subtilis* ParA protein Soj (Fig 7A) and molecular modeling using the calculated structure of the BY-kinase chimera CapA1/B2 from *Staphylococcus aureus* as template [17] suggests that the structure of the two proteins is similar (Fig 7B–7D). The accurate role of ParA in chromosome segregation remains unclear but it is proposed to interact with and assist the chromosome partitioning protein ParB during segregation of chromosomes to the daughter cells [28]. ParB specifically binds to centromere-like DNA sequences named *parS* that are located near the origin of replication [39, 40]. Therefore, to evaluate whether CpsD phosphorylation impact ParB dynamics in live cells, we constructed strains (WT, *cpsD-3YE* and *cpsD-3YF*) producing the functional fusion ParB-sfGFP [40] and we analyzed ParB dynamics by TIRF (Total Internal Reflection Fluorescence) microscopy, a technique in which only a thin section of the cell (approx. 100 nm) is excited, thus providing high axial resolution. ParB-sfGFP foci were categorized as stationary if they remained in TIRF focus during the 40 sec time-span of the experiment, while ParB-sfGFP foci appearing, disappearing or splitting were categorized as dynamic (Fig 8A and 8B). We observed that 66.9 +/- 2.8% of ParB-sfGFP were stationary in WT cells (Fig 8C). Interestingly, this fraction increased up to 80.6 +/- 2.4% in *cpsD-3YF* cells whereas it decreased down to 56.4 +/- 2.4% in *cpsD-3YE* cells (Fig 8C). These experiments thus suggest that CpsD phosphorylation could play a role in chromosome segregation by modulating ParB mobility.

### Interplay between CpsD and ParB

**Fig 9:**
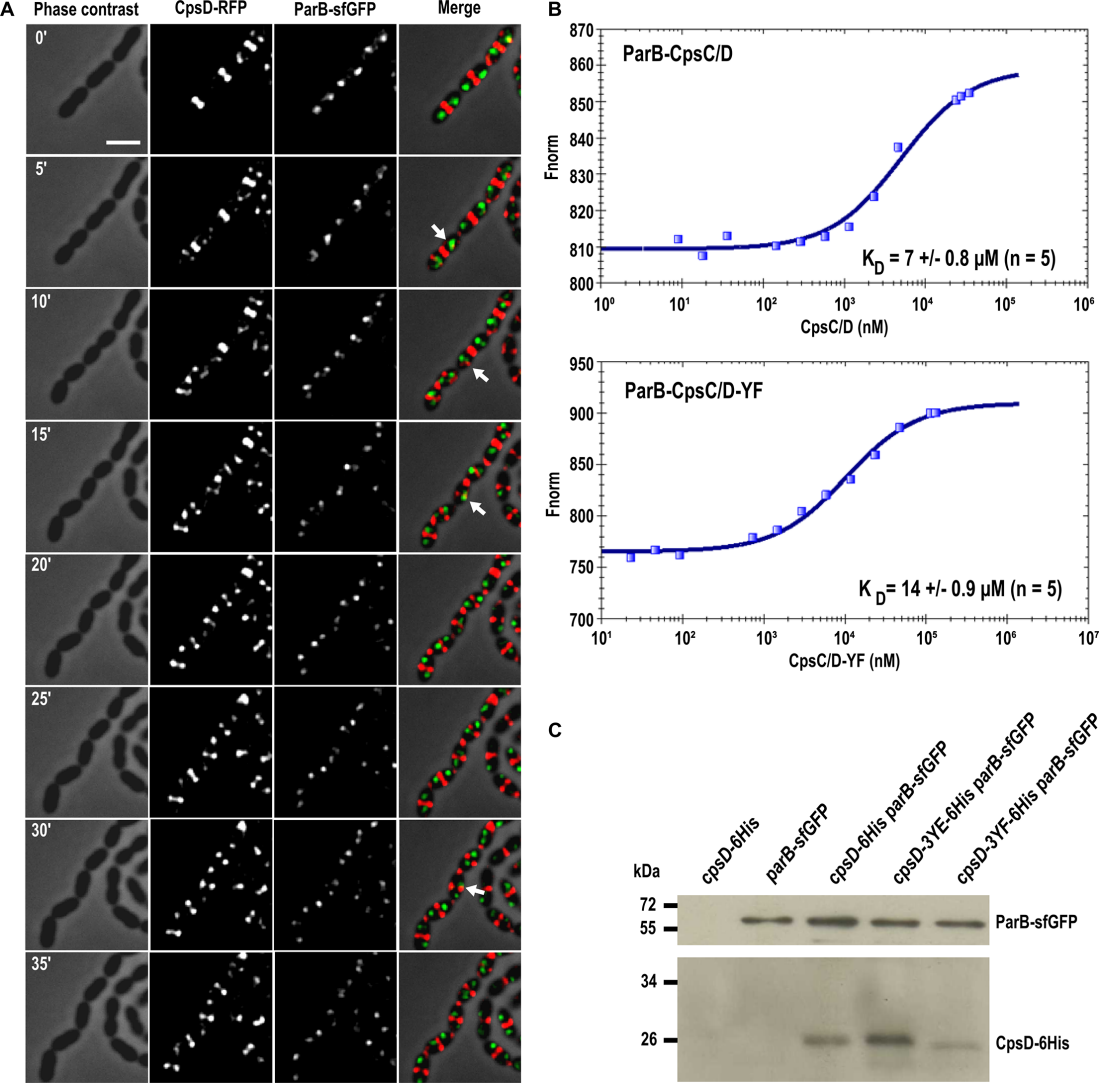
Interplay between CpsD and ParB. (A) Fluorescence time-lapse microscopy of WT cells producing CpsD-RFP and ParB-sfGFP grown in C+Y medium at 37°C. Stills are from S1 Movie. Phase contrast, RFP, GFP as well as overlays are shown. Temporal colocalization of ParB and CpsD at the division site is indicted with arrows. Scale bar, 2 μm. (B) Analysis of the interaction between ParB and the chimeras CpsC/D and CpsC/D-YF. Affinity measurements by Microscale Thermophoresis of labeled ParB binding to increasing concentrations of CpsC/D chimera. Fnorm (normalized fluorescence = fluorescence after thermophoresis / initial fluorescence) is reported on the Y axis and ligand concentrations on the x-axis are plotted in nM. Measures are represented by blue dots and fitted curves by dark blue lines. The average affinity constant of five independent experiments is indicated. (C) Immunoprecipitation of CpsD-6His with ParB-sfGFP in *cpsD-6His*, *parB-sfGFP*, *cpsD-6His parB-sfGFP*, *cpsD-3YE-6His parB-sfGFP* and *cpsD-3YF-6His parB-sfGFP* strains using anti-GFP antibodies. Samples were analyzed by immunoblotting using anti-GFP (upper panel) to check that the same amount of ParB was loaded or anti-6His-tag (lower panel) antibodies to determine the presence of co-immunoprecipitated CpsD-6His. As controls, CpsD and ParB are not detected in cells expressing either CpsD-6His but not ParB-sfGFP (*cpsD-6His* strain) or conversely ParB-sfGFP but not CpsD-6His (*parB-sfGFP* strain), respectively. Faster migration of CpsD-3YF-6His could reflect either N-terminal degradation or modified electrophoretic behavior of CpsD when non-phosphorylated.

The latter observation, combined with the fact that CpsD is homologous to ParA-type proteins, prompted us to analyze the timing of CpsD and ParB localization in living cells. To do so, we constructed a double-labeled strain expressing both ParB-sfGFP and CpsD-RFP and performed time-lapse-microcopy (Fig 9A). As shown in Fig 9A and S1 Movie, and consistent with previous observations [40], ParB-sfGFP localizes as single foci at cell equators (future division site). In these predivisional cells, CpsD-RFP did not co-localize with ParB and was exclusively detected at the current division site (Fig 9A). As the cell cycle progresses, CpsD-RFP displayed a dual localization pattern; the fusion protein remained at the division site until cell constriction was completed, but also localized at the future-division site where it co-localizes for a short time with ParB (arrows in Fig 9A). Then, as the new cell cycle began, the *oriC*-localized ParB-sfGFP moved, due to chromosome segregation, toward the daughter cell equator while CpsD-RFP remained at the division site. This transient co-localization of CpsD with ParB suggests that the two proteins might interact physically. To investigate this, we applied the approach commonly used for the purification of BY-kinases from Firmicutes: we purified the active and fully autophosphorylated CpsC/D chimera in which the cytoplasmic C-terminal end of CpsC is fused to the N-terminal end of CpsD (Fig 1C and S7A and S7B Fig). As shown in S7A Fig, ParB and the non-phosphorylatable CpsC/D-YF chimera were also purified to homogeneity. Then, microscale thermophoresis was used to determine the binding affinity of the ParB-CpsC/D complex. While no binding of ParB could be detected with the BSA (Bovine Serum Albumin) control (S7C Fig), an affinity constant of 7 ± 0.8 μM (n = 5) was obtained for the ParB-CpsC/D complex (Fig 9B). A two-fold higher affinity constant was calculated for the ParB-CpsC/D-YF complex (14 ± 0.9 μM (n = 5) suggesting that CpsD autophosphorylation influenced the interaction between ParB and CpsC/D. Then, we investigated the interaction between ParB and CpsD *in vivo*. For that, we constructed strains producing ParB-sfGFP together with either CpsD, CpsD-3YE or CpsD-3YF fused to a 6his-tag. We checked that fusions were produced at similar levels (S7D Fig). After immunoprecipitation of ParB-sfGFP, the presence of CpsD variants in the immunoprecipitated fractions was probed by Western blot using anti-His antibodies (Fig 9C). Western blots were also performed using anti-GFP antibodies to confirm that similar amounts of ParB-sfGFP were loaded. We observed that all CpsD constructs co-immunoprecipitated with ParB-sfGFP. Yet, we observed that immunoprecipitation of CpsD-3YF by ParB-sfGFP was less efficient than that of CpsD or CpsD-3YE. Altogether, these observations indicate that CpsD interact with ParB and that this interaction is modulated by CpsD phosphorylation.

## Discussion

Many reports have demonstrated that BY-kinases are key regulators of extracellular polysaccharides biosynthesis and export [22, 24, 41–44]. The current model proposes that they would function as co-polymerases assisting the polymerase of the capsule assembly machinery [8]. However, the detailed mechanism by which BY-kinases control this machinery remains unknown. It is proposed that BY-kinases could serve as a molecular scaffold for the other proteins of the machinery. Phosphorylation / dephosphorylation of BY-kinases would trigger a conformational switch affecting the functioning of the other protein components of the polysaccharide assembly machinery [17]. Alternatively, BY-kinases could form a channel across the cytoplasmic membrane, large and hydrophilic enough to allow the polysaccharide polymer to cross the membrane. Indeed, BY-kinases can form a ring-shaped octamer that upon autophosphorylation dissociates to monomers [21]. Recently, Henriques and co-workers have shown that the BY-kinase CpsD of *S*. *pneumoniae* ATCC6314 localizes at the bacterial division septum suggesting that BY-kinases might also function as spatial regulators of capsular polysaccharide biosynthesis [13]. They propose that in the pneumococcus, two types of CPS assembly machinery would allow to wrap the cell in CPS. The membrane machinery without CpsC and CpsD would allow production of CPS around the cell while the septal machinery, associated with CpsC and CpsD, would specifically produce CPS at the septum. Our finding that CpsH localizes exclusively at the septum in WT cells allows us to refine this model and strongly suggests that CPS are produced only at the division septum by a single machinery (Fig 3D). This observation is further supported by the septal localization of CpsJ (S8 Fig). The capsule detected at the old cell poles in the Δ*cpsD* mutant could thus reflect accumulation of basal amount of immature capsule produced by the defective and mislocalized CPS assembly machinery. To our knowledge, this represents the first study determining the cellular site of polysaccharide synthesis and export.

In this context, what is the contribution of CpsC and CpsD to CPS production at the division septum? Our data show that the C-terminal and cytoplasmic end of CpsC is required for the interaction between CpsC and CpsD and consequently, CpsD autophosphorylation and localization at the division septum (Fig 2). These data are in agreement with the existence of a conserved activation mechanism of BY-kinase autophosphorylation. We observe that both CpsD and CpsH delocalize in cells expressing CpsC devoid of its C-terminal cytoplasmic end (*cpsC-*Δ*Cter*). Yet, CpsH also delocalizes in cells deficient for *cpsD* (Fig 3D). Collectively, these data are consistent with a sequential interaction model in which CpsC captures CpsD at the division septum allowing subsequent localization of CpsH at the septum. The absence of capsule at the division septum together with impaired polymerization of CPS in *cpsC-*Δ*Cter* and Δ*cpsD* cells (Fig 3A–3C) support this model. One should, however, note that CPS are still detected at the old cell pole in *cpsC-*Δ*Cter* and Δ*cpsD* cells (Fig 3A). This shows that the capsule assembly machinery is still able to export some polysaccharides at the surface of cells in these genetic backgrounds. This observation implies that some polysaccharide subunits produced in the cytoplasm are flipped across the membrane by the flippase CpsJ but not properly polymerized by CpsH. Supporting this, cells deficient for *cpsJ* do not produce CPS [45]. This indicates that CpsC and CpsD are unlikely to function themselves as the exporter as previously suggested by Henriques and co-workers and our previous work [13, 21]. More likely, CpsC together with CpsD capture the CPS assembly machinery at the division site and trigger CPS export and polymerization by the flippase CpsJ and the polymerase CpsH, respectively.

One may also wonder how CPS produced at the division site covers the whole cell. An interesting possibility lies in the mode of cell division and elongation of the pneumococcus. Contrary to rod-shaped bacteria, the pneumococcus does not perform lateral synthesis of peptidoglycan. Peptidoglycan is produced at mid-cell and serves both for cell elongation and division, resulting in its characteristic ovoid morphology [46]. More precisely, it was demonstrated that ongoing peptidoglycan synthesis pushes the previous synthesized peptidoglycan leading mechanically to cell elongation and then formation of the new cell pole [47]. Considering that CpsA localizes at midcell and likely ligates CPS to the cell wall [10, 48, 49], CPS concurrently produced with peptidoglycan at mid-cell could be shuttled by peptidoglycan as the cell elongates and constricts. Because CPS are required to avoid pneumococcus recognition by the host complement and immune systems, this would provide a very simple mechanism to conceal and cover the complete surface of the cell. To test this hypothesis, the generation of fluorescent CPS precursors might be useful, similar to the approaches used to image peptidoglycan synthesis [50].

An important feature of BY-kinases regulation of CPS synthesis and export is their phosphorylation on several tyrosine residues grouped in a C-terminal motif termed “tyrosine cluster” [19]. Here, we observe that the *cpsD-3YF* strain displays slightly reduced production of CPS (Fig 3C). This finding is consistent with previous observations made in *E*. *coli* and *S*. *pneumoniae* [20, 22, 23, 25]. In addition, we observe that the localization of CpsD remains unchanged whatever its phosphorylation state (Fig 5A). This suggests that CpsD autophosphorylation is not crucial for the activity of CPS assembly machinery *per se*. However, and strikingly, expression of CpsD-3YF hinders CPS production at mid-cell, alter CpsH septal localization and results in an elongated cell phenotype (Figs 3 and 4). Altogether, these data suggest that CpsD phosphorylation would coordinate CPS production with the pneumococcal cell cycle. In this context, the presence of permanently non-phosphorylated CpsD (CpsD-3YF) at the division site would interfere with cell constriction, leading to elongated cells. Our finding that the non-septal localization of CpsD-3YF (due to deletion of the CpsC C-terminus) (Fig 5A) suppresses the elongated phenotype of the *cpsD-3YF* mutant cells (Fig 4), together with the presence of non-constricted septa in *cpsD-3YF* cells (Fig 5B), support this hypothesis.

Our data also show that the *cpsD-3YF* elongated cells display nucleoid defects (Fig 6). Interestingly, BY-kinases have been grouped with ParA and MinD proteins in the same protein superfamily on the basis of sequence similarity-based clustering [27]. ParA and MinD are involved in chromosome segregation and positioning of the Z-ring at mid-cell, respectively [51]. The first structure of the BY-kinase CapB from *S*. *aureus* was solved by molecular replacement using the structure of MinD from *Pyrococcus horikoshii*, and the two structures are highly similar [17]. Interestingly, the pneumococcus lacks ParA and MinD proteins [51]. On this basis, it is tempting to speculate that CpsD could share some functional properties with either ParA or MinD. Positioning of the Z-ring at mid-cell has been recently elucidated in the pneumococcus and it relies on the protein MapZ [47, 52]. Together with our finding that Z-rings are recruited to each non-constricted septum of *cpsD-3YF* cells, it is unlikely that CpsD contributes to division site selection as is the case for MinD (Fig 5C). However, it would be interesting to see whether the CpsD-phosphorylation mutants (YE/YF), by affecting CPS production, also cause an imbalance in PG precursor levels and hence cell division defects. By contrast, the structural similarity between CpsD and ParA proteins (Fig 7) suggests that CpsD could behave as a ParA-like protein even if we did not detect any DNA-binding for CpsD (S9 Fig). The accurate role of ParA in chromosome segregation remains unclear but it is proposed to interact with and assist ParB during chromosome segregation [28]. Unencapsulated strains of pneumococcus do not possess CpsD, thus suggesting that CpsD is unlikely to represent an authentic ParA protein involved in chromosome segregation. However, Δ*cps* unencapsulated cells are also significantly shorter (Fig 4), suggesting that the production of the large CPS structure may require additional checkpoints to ensure correct chromosome segregation and cell division. Several studies have reported that ParA-like proteins are actually involved in protein localization in connection with the cell cycle [53]. For instance, ParC and PpfA facilitate polar localization and segregation of chemotaxis proteins in *Vibrio cholerae* and *Rhodobacter sphaeroides*, respectively [54, 55]. Another example is the ParA-like protein FlhG that is required for the polar localization of flagellar assembly factors [56]. Considering the CPS and nucleoid defects of *cpsD-3YF* cells, together with the timing of localization of CpsD and ParB and the ability of CpsD to interact with ParB (Figs 6 and 9), it is tempting to speculate that a crosstalk between the ParA-like CpsD protein and ParB could coordinate septal CPS production with the cell cycle. Our finding that CpsD phosphorylation modulates the stability of the complex encompassing CpsD and ParB (Fig 9B and 9C) further suggests that CpsD phosphorylation constitutes a system for signaling the CPS synthesis status to chromosome segregation and ensuring that daughter cells are properly wrapped in CPS.

**Fig 10:**
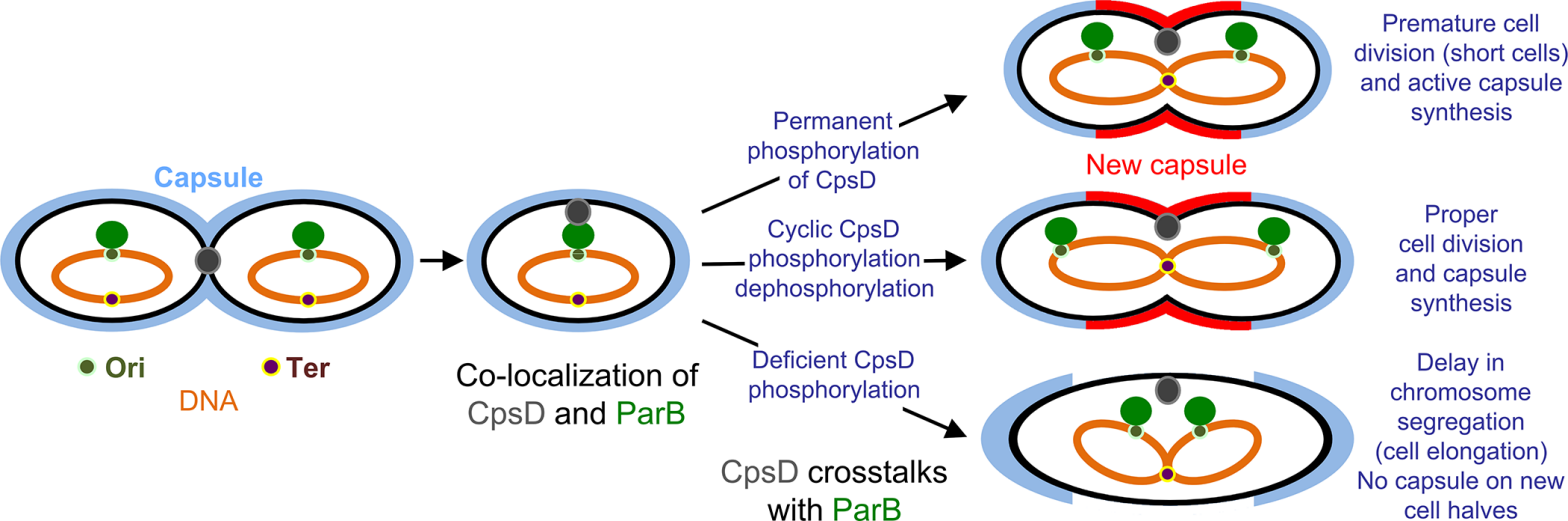
Model for CpsD-mediated coordination of CPS synthesis with the cell cycle. In newborn WT cells, CpsD localizes at the ongoing division site and ParB localizes at the cell equator (future cell division site). As the cell cycle progresses, CpsD moves towards the cell equator and localizes transiently together with ParB at the cell equator. Then, ParB bound to *parS* sites located near the origin of replication (*Ori*) moves toward the new cell equator as the chromosomes segregate. This timing is modulated by the CpsD phosphorylation state. In absence of CpsD phosphorylation, the crosstalk between CpsD and ParB is hampered leading to reduced ParB mobility and delayed chromosome segregation. This delay induces cell constriction defects and thus cell elongation. By contrast, when phosphorylated, CpsD efficiently signals that the CPS assembly machinery is functional to chromosome segregation *via* ParB inducing premature cell constriction. In WT cells, the intermediate situation is observed due to the presence of both phosphorylated and non-phosphorylated CpsD. ParB and CpsD are indicated by green and grey circles, respectively. Brown and khaki dots indicate *Ter* and *Ori* region. The chromosome is shown in orange. Capsule is in light blue (mother cell) and red (daughter cell).

Collectively, these data fit into the model presented in Fig 10. ParB localizes first at the division site, rapidly followed by CpsD before chromosome segregation starts. When CpsD is not phosphorylated, reflecting that CpsD does not hydrolyze ATP and that the CPS assembly machinery is switched off, chromosome segregation is delayed due to reduced ParB mobility. By contrast, when the CPS assembly machinery is switched on, CpsD hydrolyzes ATP and autophosphorylates. Phosphorylation of CpsD then favors the mobility of ParB and chromosome segregation. CPS would thus cover the new cell halves as the cell elongates and constricts. Consistent with this model, *cpsD-3YE* cells are significantly shorter in length (Fig 4) reflecting that cell constriction likely occurs prematurely due to an enhanced crosstalk between CpsD and ParB.

Finally, our data question the raison d’être of such a phospho-regulatory process. While *cpsD-3YE* cells are slightly shorter in length, they divide properly and are covered by CPS (Fig 3A). An interesting and promising hypothesis concerns the life-style of the pneumococcus. CPS are required during infection for protection against the human immune system but they are also disadvantageous because of their inhibitory effects on adherence to the host cell [3, 6, 57, 58]. One can speculate that the presence of a regulatory mechanism based on CpsD phosphorylation coordinating CPS production with the cell cycle would allow modulating capsule production to satisfy optimal colonization and dissemination.

## Materials and Methods

### Strains and growth conditions

*S*. *pneumoniae* strains were cultivated at 37°C in Todd-Hewitt Yeast (THY) broth (Difco) or in C+Y medium [59]. *S*. *pneumoniae* mutants were constructed by transformation in D39 as previously described [60], using precompetent cells treated at 37°C with synthetic competence stimulating peptide 1 (CSP1) to induce competence. Transformants were selected on THY-agar or Columbia agar supplemented with either 3% (vol/vol) defibrinated horse blood or 2% defibrinated sheep blood and containing the appropriate antibiotic (streptomycin 200 μg.mL^-1^, kanamycin 250 μg.mL^-1^, spectinomycin 100 μg.mL^-1^, chloramphenicol 2 μg.mL^-1^). Strains complemented with *cpsC* or *cpsD* at the ectopic *amiF/treR* locus under the control of the maltose inducible promoter P_M_ [31] were grown in C+Y medium containing 20% maltose. The *E*. *coli* XL1-Blue strain was used as a host for cloning. *E*. *coli* BL21 (DE3) strain was used as a host for overexpression. Luria-Bertani (LB) broth and agar supplemented with appropriate antibiotic (tetracycline 15 μg.mL^-1^, ampicillin 100 μg.mL^-1^) were used for routine growth at 37°C. Strains used in this study are listed in S1 Table.

### Allelic replacement mutagenesis

To construct pneumococcus mutants (gene deletions, *gfp*/*rfp*/*sfgfp* fusions or site-directed mutagenesis), we used a two-step procedure based on a bicistronic *kan-rpsL* cassette called Janus [61], except for allelic replacement of *parB* by *parB-sfGFP* and *hlpA* by *hlpA-RFP*, where we used a one-step procedure with a spectinomycin or chloramphenicol resistance marker, respectively [36, 40]. Throughout this study, gene mutagenesis or fusion with fluorescent protein were constructed at each native chromosomal locus, expressed under the control of the native promoter and represented the only source of protein. Full description of primers used for the construction of strains is provided in S2 Table. The genes encoding monomeric sfGFP, monomeric GFP and RFP were from [33, 62, 63], respectively.

### Construction of plasmids

DNA fragments coding for CpsC, CpsC-ΔCter, CpsD and ParB were obtained by PCR using chromosomal DNA from *S*. *pneumoniae* D39 as template. For the chimera CpsC/D, we used DNA from strain TIGR4. Oligonucleotides used are described in S2 Table. The chimera DNA fragment was constructed by fusion of fragments obtained using primer pairs I-III and IV-II. The obtained DNA fragment was cloned between the *Bam*HI and *Hind*III cloning sites of the pQE30 plasmid (Qiagen). *parB* was cloned between the *Nde*I and *Pst*I cloning sites of the pT7.7 plasmid [64]. To construct plasmids for yeast two-hybrid, the PCR DNA fragments were digested by *Eco*RI and *Pst*I and ligated either into pGAD-C1 or pGBDU-C1 vectors [65]. The nucleotide sequences of all DNA fragments were checked to ensure error-free amplification. Plasmids and primers used in this study are listed in S1 and S2 Tables, respectively.

### Protein purification

Recombinant plasmids overproducing the chimera CpsC/D and ParB were transformed into BL21 (DE3) *E*. *coli* strain. The transformants were grown at 37°C until the culture reached an OD_600_ = 0.5. Expression was induced by adding IPTG to a final concentration of 0.5 mM and incubation was continued for 3 h. After 3 h culture at 37°C, cells were harvested and resuspended in buffer A (Tris-HCl 50 mM, pH 7.5; NaCl 300 mM; DTT 1 mM; imidazole 10 mM; glycerol 10%) containing 10 mg.L^-1^ of lysozyme and 6 mg.L^-1^ of DNase I and RNase A and sonicated. After centrifugation at 15000 g for 30 min, the supernatant was applied to a Ni-NTA agarose column (Qiagen) and extensively washed with buffer A supplemented with 30 mM imidazole. Samples were eluted with buffer A supplemented with 300 mM imidazole. The fractions corresponding to the pure protein were pooled and dialyzed against the following buffer: HEPES 50 mM, pH 7.5; NaCl 100 mM; DTT 1 mM; MgCl_2_ 1 mM; glycerol 10%. The protein concentrations were determined using a Coomassie Assay Protein Dosage Reagent (Uptima) and aliquots were stored at -80°C.

### Co-immunoprecipitation

Cultures of *S*. *pneumoniae* cells were grown at 37°C in THY medium until OD_550_ = 0.4. Cells pellets were incubated at 4°C for 15 min in buffer B (Tris-HCl 50 mM, pH 8.0; NaCl 150 mM; MgCl_2_ 5 mM) containing 0.1 mg.mL^-1^ of lysozyme, 800 units of mutanolysine and 0.2 mg.mL^-1^ of DNase I and RNase A and sonicated. After centrifugation, the supernatant was incubated with the GFP-Trap resin suspension (Chromotech). Resin was washed according to the manufacturer’s instructions. Protein-bounded GFP-Trap resins were eluted with Laemmli buffer at 95°C for 10 min and analyzed by SDS-PAGE followed by an immunoblot directed against 6-Histidines tag or GFP.

### Yeast two-hybrid

The yeast two-hybrid phenotypic assays were performed as described previously [66]. Briefly, genes encoding for CpsD, CpsC and CpsC-ΔCter were fused to either the activating domain of Gal4 or the DNA-binding domain of Gal4. Resulting plasmids were inserted in yeast haploid cells and interactions were screened for ability to grow on the selective medium.

### Immunoblot analysis

*In vitro* CpsC/D autophosphorylation and *in vivo* phosphorylated proteins in crude extract of *S*. *pneumoniae* were immunodetected using mouse anti-phosphotyrosine monoclonal antibody PY-20 (Sigma-Aldrich) at 1/2000. Detection of GFP fusions was performed using a rabbit anti-GFP polyclonal antibody (AMS Biotechnology) at 1/10000. Detection of Enolase was performed using a rabbit anti-enolase polyclonal antibody at 1/50000 [32]. Proteins fused to a 6-histidines tag were detected using a mouse anti-6His monoclonal antibody (Sigma-Aldrich) at 1/1500. Detection of capsular polysaccharides was performed using a rabbit anti-serotype 2 CPS polyclonal antibody (Statens serum Institute) at 1/2000. A goat anti-rabbit or anti-mouse secondary polyclonal antibody horseradish peroxidase (HRP) conjugated (Biorad) was used at 1/5000 to reveal immunoblots.

### Preparation and analysis of CPS

CPS were prepared as previously described with minor modifications [67]. Briefly, *S*. *pneumoniae* cultures were grown until OD_550_ = 0.3 and cells were harvested by centrifugation at 14,000xg for 20 min at 4°C. Pellets were then washed once with PBS and resuspended in buffer A (Tris-HCl 50 mM, pH 7,4; sucrose 20%; MgSO_4_ 50 mM) at 1/100 of the original culture volume. The solution was then added with 400 units of mutanolysin and 6 μg of DNase and RNase per milliliter of solution and incubated overnight at RT. After centrifugation at 16,000xg for 20 min at 4°C, pellets were resuspended in the same volume of buffer A. 10 μL of the mixture were then mixed with 5 μL of buffer B (Tris-HCl 50 mM, pH 8.0; EDTA 50 mM; Tween20 0.5%; Triton X100 0.5%) and 20 μg of proteinase K, incubated 30 min at 37°C and analyzed by immunoblotting as previously described [68].

### Molecular modeling

Protein sequence alignments were obtained using ClustalW and ESPript3 [69, 70]. Predicted secondary structures of CpsC/D and the *B*. *subtilis* Soj were made using Jpred3 [71]. The three-dimensional model of the chimera CpsC/D, based on the structure of the CapA1/B2 protein (PDB code 3BFV) was built using I-Tasser [72]. The visualization of 3D-molecules was performed using PyMOL (Schrödinger).

### Microscale thermophoretic analysis

Microscale thermophoresis was used to test the interaction of ParB with the chimeras CpsC/D and CpsC/D-YF [73]. BSA (Bovine Serum Albumin) was used as negative control. Binding experiments were carried out with a Monolith NT.115 Series instrument (Nano Temper Technologies GMBH). ParB was labeled with the red dye NT-647. Briefly, 4 μl of sample containing 100 nM of labeled ParB and increasing concentrations of CpsC/D (from 4 nM to 92 μM) or BSA (from 5 nM to 180 μM) were loaded on K003 Monolith NT.115 hydrophilic treated silicon capillaries and thermophoresis was measured for 30 s. Each measurement was made in triplicates. Experiments were carried out at 25°C in MST optimized buffer (50 mM Tris-HCl, 150 mM NaCl, 10 mM MgCl_2_, 0.05% Tween-20). Analysis was performed with the Monolith software. Affinity K_D_ was quantified by analyzing the change in normalized fluorescence (Fnorm = fluorescence after thermophoresis/initial fluorescence) as a function of the concentration of the titrated CpsC/D protein. The fraction of ParB bound was plotted against the concentration of CpsC/D.

### Microscopy techniques

Microscopy was performed on exponentially growing cells (OD_550_ = 0.3). For *in vivo* immunofluorescence microscopy, cells were mixed with an rabbit anti-serotype 2 CPS polyclonal antibody (Statens Serum Institute) at 1/1000, washed several times with THY at 37°C and then incubated with an anti-rabbit DyLight-549 (Jackson ImmunoResearch) at 1/2000. After a last wash with PBS, CPS were imaged. For DAPI, 10 μL of *S*. *pneumoniae* cell culture were mixed with 1 μL of DAPI at 2 mg.mL^-1^ (Molecular Probes) and incubated 5 min at room temperature. GFP, RFP and sfGFP fusions were visualized by fluorescence microscopy. Slides were visualized with a Zeiss AxioObserver Z1 microscope fitted with an Orca-R2 C10600 charge-coupled device (CCD) camera (Hamamatsu) with a 100× NA 1.46 objective. Images were collected with axiovision (Carl Zeiss), deconvolved with ImageJ (http://rsb.info.nih.gov/ij/) and analyzed with Coli-Inspector (detection was approved manually) running under the plugin ObjectJ (http://simon.bio.uva.nl/objectj/) to measure CPS fluorescence intensity or generate fluorescent intensity linescans sorted to cell length and MicrobeTracker suite [74] extended by custom MATLAB routines to generate cell length distribution histograms. Cell lengths were determined using MicrobeTracker. Images are representative of experiments made in triplicate.

Fluorescence time-lapse microscopy was performed on a DV Elite (Applied Precision) with a sCMOS camera using SSI Solid State Illumination (Applied Precision) through a 100× oil immersion objective (phase contrast), essentially as described before [36, 75]. During the experiment, cells were incubated on a slide of C+Y agarose and kept at 37°C in a temperature controlled chamber. Phase contrast and fluorescent (GFP and RFP) images were acquired every 5 min. Images were processed using softWoRx 5.5 (Applied Precision) and ImageJ.

For TEM, *S*. *pneumoniae* cells (wild type and mutants) were collected, centrifuged and pre-fixed 20 min on ice with the fixing mix (cacodylate 0.1 M, pH 7.4; glutaraldehyde 5%; lysine-HCl 0.075 M; ruthenium red 0.075%). After washing, cells were fixed overnight at 4°C with fixing mix without lysine-HCl. Cells were took onto 2% agarose and post-fixation with 1% osmium tetroxide in cacodylated buffer added to ruthenium red was carried out for 1 h at room temperature. These fixed cells were dehydrated using a graded series of ethanol and embedded in LR-White at 60°C for 48 h. Ultrathin sections (60 nm) were obtained using a Leica UC7 microtome and were counter-stained with uranyl acetate and lead citrate (Reichert Ultrostainer, Leica). Samples were examined with a Phillips CM120 transmission electron microscope equipped with a Gatan Orius SC200 CCD camera.

TIRF microscopy was performed as described before [36]. Cells were grown in C+Y until mid-exponential phase prior to examination on a DV Elite (Applied Precision) with a sCMOS camera using 50 mW laser illumination (488 nm and 561 nm) through a 100× oil 1.49 NA TIRF objective. Cells were imaged every 10 seconds for 40 seconds. The GFP foci in the cells were classified as stationary or dynamic (splitting, appearing or disappearing foci) using intensity line scans at different time points in Image J. Fluorescence foci were classified as “splitting” when one fluorescence peak splits in two peaks during the time span of the experiment and foci were classified as “disappearing” when the signal of a fluorescence peak was reduced >90% during the time span of the experiment.

## Supporting Information

S1 Fig Non-polarity of the markerless insertion of *cpsC-*Δ*Cter* and deletion of *cpsD*. (A) Expression of CpsD fused to GFP in WT and cpsC-Δ*Cter strains*. CpsD-GFP was detected using anti-GFP antibodies (α-GFP). To estimate the relative quantity of CpsD-GFP in crude extract and to compare the two lanes, we used the enolase as an internal standard. The enolase was detected using specific antibodies (α-Eno) [32] and is presented in the lower panel. The analysis showed that the two strains synthesized similar amounts of CpsD-GFP. (B) Detection of CPS in living complementation cells Δ*cpsD* P_*M*_ *-cpsD* or *cpsC-*Δ*Cter* P_*M*_ *-cpsC* by immunofluorescence. CPS were immunodetected with a rabbit anti-serotype 2 CPS polyclonal antibody. CPS fluorescent signal (red, left panels) and overlays (right panels) between phase contrast and CPS fluorescence images are shown. Scale bar, 2 μm. (C) Detection of cell-associated CPS in the WT strain and complemented Δ*cpsD* P_*M*_ *-cpsD* or *cpsC-*Δ*Cter* P_*M*_ *-cpsC* strains. The immunoblot was probed with a rabbit anti-serotype 2 CPS polyclonal antibody.
(TIF)

S2 Fig Expression of CpsH-sfGFP in WT and mutant strains. The Western immunoblot was probed with anti-GFP antibodies (α-GFP) to determine CpsH-sfGFP expression in WT, *cpsD-3YF cpsH-sfGFP*, *cpsD-3YE cpsH-sfGFP*, Δ*cpsD cpsH-sfGFP* and *cpsC-*Δ*cter cpsH-sfGFP* cells. To estimate the relative quantity of proteins in crude extract and to compare the different lanes, we used the enolase as an internal standard (α Eno). The enolase was detected using specific antibodies as described in [32] and is presented in the lower part of the figure.
(TIF)

S3 Fig Analysis of WT cells expressing either CpsD-GFP or CpsH-sfGFP. (A) Growth curves of WT strains expressing either CpsD-GFP (upper panel) or CpsH-sfGFP (lower panel) as the only source of CpsD or CpsH from their endogenous chromosomal locus grown in THY medium at 37°C. The OD_550_ was read automatically every 10 min. (B) Detection of CPS in living *cpsD-GFP* and *cpsH-sfGFP* cells. CPS were immunodetected with a rabbit anti-serotype 2 CPS polyclonal antibody. CPS fluorescent signal (red, left panels) and overlays (right panels) between phase contrast and CPS fluorescence images are shown. Scale bar, 2 μm. (C) Detection of cell-associated CPS produced by WT, *cpsD-GFP* and *cpsH-sfGFP* strains. The immunoblot was probed with a rabbit anti-serotype 2 CPS polyclonal antibody. (D) Quantification of the total CPS fluorescent signal in living WT, *cpsD-GFP* and *cpsH-sfGFP* cells. n indicates the number of cells analyzed and standard deviation from the fluorescence of the n cells is indicated with error bars.
(TIF)

S4 Fig Expression of WT and mutated CpsD-GFP in WT and *cpsC-*Δ*Cter* strains. Expression of CpsD-GFP, CpsD-3YF-GFP, CpsD-3YE-GFP in WT cells and CpsD-3YF-GFP in *cpsC-*Δ*Cter cells*. WT or mutated CpsD-GFP was detected using anti-GFP antibodies (α-GFP). To estimate the relative quantity of CpsD-GFP in crude extract and to compare the lanes, we used the enolase as an internal standard (α-Eno). The enolase was detected using specific antibodies [32] and is presented in the lower panel.
(TIF)

S5 Fig Transmission electron microscopy images. Transmission electron micrographs of WT (left column), *cpsD-3YE* (middle column) and *cpsD-3YF* (right column) strains. Scale bar, 1 μm.
(TIF)

S6 Fig Origin-to-terminus ratios in WT and cpsD-3YF cells. The CpsD-3YF mutation does not lead to differences in origin-to-terminus ratio. WT and CpsD-3YF were grown to OD_600_ = 0.15 for isolation of genomic DNA. The box-plots show the origin-to-terminus ratio as determined by qPCR. The data were analyzed by Monte Carlo simulations. There are no significant difference between the wild-type and the CpsD-3YF strain (*p*-value > 0.05). Whiskers represent the 10th and 90th percentiles, and the circles represent the outliers from Monte Carlo simulation data. qPCR, data analysis and the statistical test was done as described previously in [38].
(TIF)

S7 Fig Protein purification and interaction control. (A) Purification of ParB and chimeras CpsC/D and CpsC/D-YF. Proteins were overproduced in *E*. *coli* BL21 as 6his-tagged fusion proteins. After purification using a Ni-NTA agarose resin, they were analyzed by SDS-PAGE and coomassie blue staining. (B) Autophosphorylation of the chimera CpsC/D. 0.1 μg of purified CpsC/D from *E*. *coli* cells were incubated in the presence (+) or absence (-) of 5 mM ATP for 30 min at 37°C and analyzed by SDS-PAGE and electro-transferred onto a PVDF membrane. CpsD phosphorylation was then immunodetected using mouse anti-phosphotyrosine monoclonal antibody PY-20. (C) Affinity measurements by Microscale Thermophoresis of labeled ParB binding to increasing concentrations of BSA. No binding could be detected. (D) Expression level of the different forms of CpsD-6His and ParB before co-immunoprecipitation in *cpsD-6His parB-sfGFP* (lane 1), *cpsD-3YE-6His parB-sfGFP* (lane2) and *cpsD-3YF-6His parB-sfGFP* (lane 3) cells.
(TIF)

S8 Fig Localization of CpsJ-sfGFP in WT cells. Phase contrast (left), GFP fluorescent signal (middle) and overlays (right) between phase contrast (red) and GFP (green) images are shown. The map of CpsJ-sfGFP fluorescence profiles of 360 cells sorted according to their length is presented. The total integrated fluorescence of each cell is plotted as function of its cell length (y-axes) and all cells are plotted with increasing cell length from left to right (x-axes). Scale bar, 2 μm.
(TIF)

S9 Fig Electrophoretic mobility shift assay of Soj and CpsC/D DNA binding. Soj from *Bacillus subtilis* was purified as described in [76]. Increasing amounts of Soj and CpsC/D were incubated with pUC18 DNA (20 fmol) in the presence of 1 mM ATP and run on a 0,6% agarose gel as previously described for Soj [76, 77]. Protein concentrations were as follow: Lane 1, no protein; Lane 2, SoJ 20 pmol; Lane 3, Soj 40 pmol; Lane 4, Soj 80 pmol; Lane 5, Soj 160 pmol; Lane 6, Soj 240 pmol; Lane 7, CpsC/D 20 pmol; lane 8, CpsC/D 40 pmol; Lane 9, CpsC/D 80 pmol; Lane 10, CpsC/D 160 pmol; Lane 11, CpsC/D 240 pmol. CpsC/D fails to bind DNA whereas Soj does.
(TIF)

S1 Movie Time-lapse analysis of ParB-sfGFP and CpsD-RFP in wild-type cells. The video shows an overlay of GFP (green), RFP (red) and phase-contrast (gray) images.
(MP4)

S1 Table Strains and plasmids. (PDF)

S2 Table List of primers. (PDF)
