# Supplementary figures and images for: Autophosphorylation of the Bacterial Tyrosine-Kinase CpsD Connects Capsule Synthesis with the Cell Cycle in Streptococcus pneumoniae
Source: PLoS Genet. 2015 Sep 17;11(9):e1005518. doi: 10.1371/journal.pgen.1005518 (PMC4574921; doi:10.1371/journal.pgen.1005518)

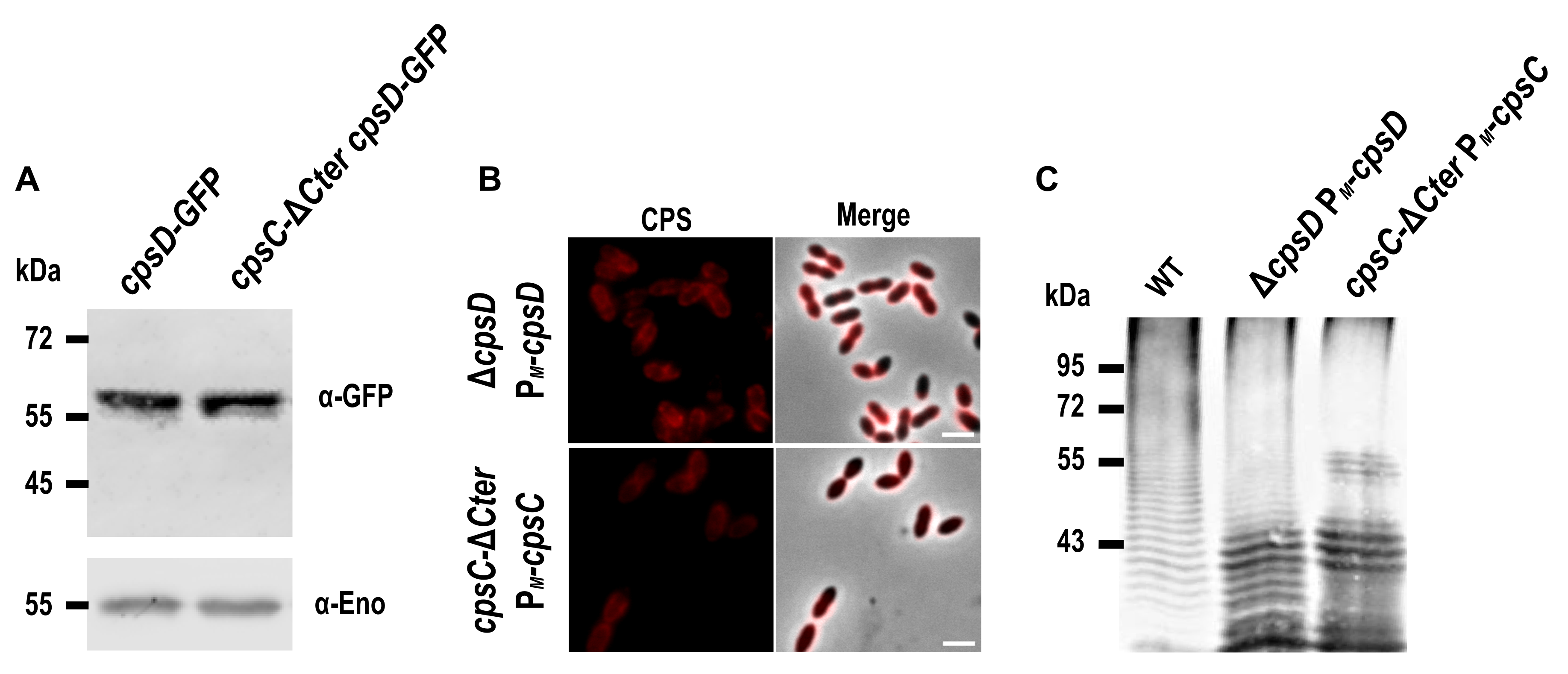

Supplement: S1 Fig — (A) Expression of CpsD fused to GFP in WT and cpsC-ΔCter strains. CpsD-GFP was detected using anti-GFP antibodies (α-GFP). To estimate the relative quantity of CpsD-GFP in crude extract and to compare the two lanes, we used the enolase as an internal standard. The enolase was detected using specific antibodies (α-Eno) [32] and is presented in the lower panel. The analysis showed that the two strains synthesized similar amounts of CpsD-GFP. (B) Detection of CPS in living complementation cells ΔcpsD PM -cpsD or cpsC-ΔCter PM -cpsC by immunofluorescence. CPS were immunodetected with a rabbit anti-serotype 2 CPS polyclonal antibody. CPS fluorescent signal (red, left panels) and overlays (right panels) between phase contrast and CPS fluorescence images are shown. Scale bar, 2 μm. (C) Detection of cell-associated CPS in the WT strain and complemented ΔcpsD PM -cpsD or cpsC-ΔCter PM -cpsC strains. The immunoblot was probed with a rabbit anti-serotype 2 CPS polyclonal antibody. (TIF) [file pgen.1005518.s001.tif]

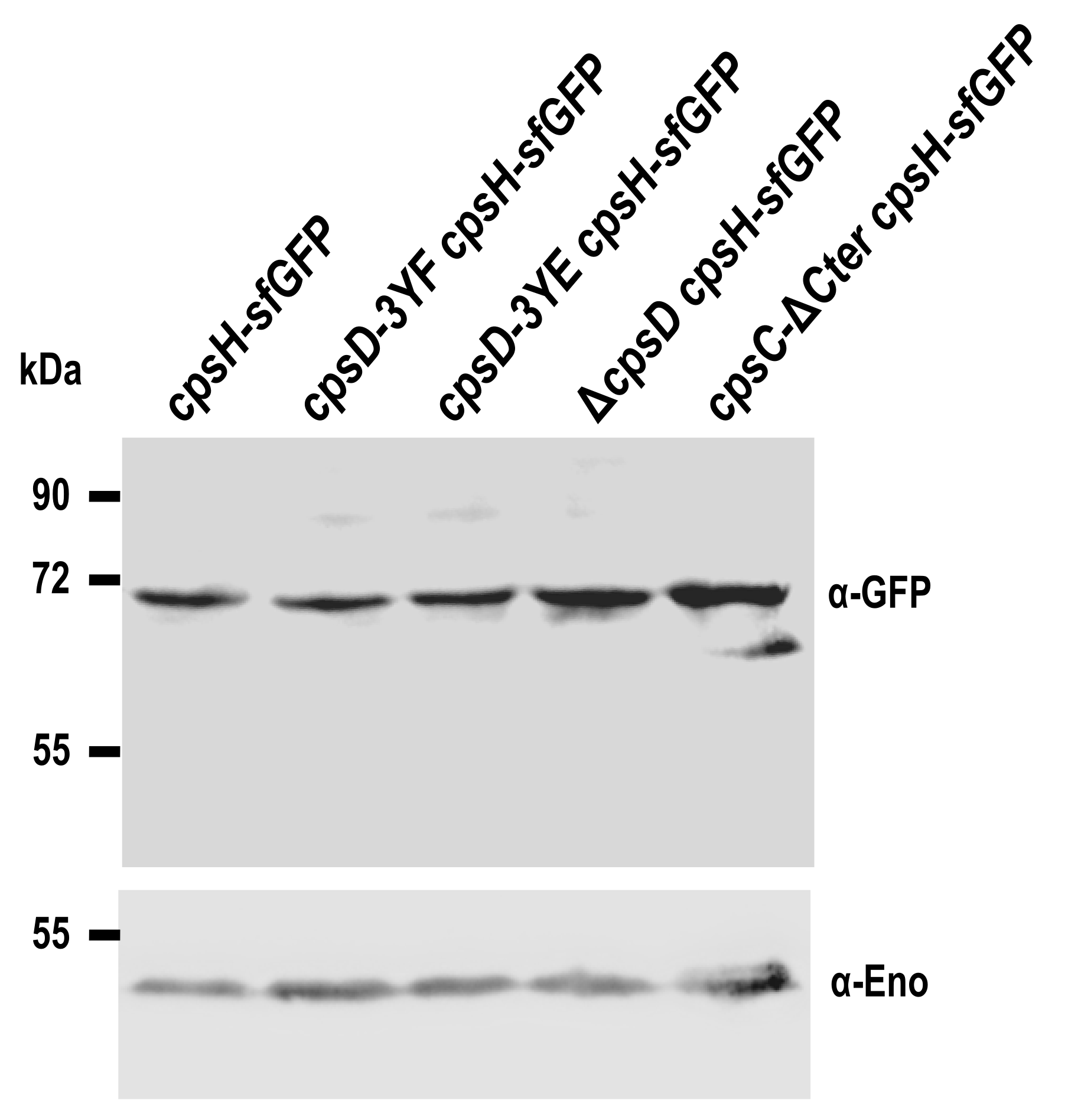

Supplement: S2 Fig — The Western immunoblot was probed with anti-GFP antibodies (α-GFP) to determine CpsH-sfGFP expression in WT, cpsD-3YF cpsH-sfGFP, cpsD-3YE cpsH-sfGFP, ΔcpsD cpsH-sfGFP and cpsC-Δcter cpsH-sfGFP cells. To estimate the relative quantity of proteins in crude extract and to compare the different lanes, we used the enolase as an internal standard (α Eno). The enolase was detected using specific antibodies as described in [32] and is presented in the lower part of the figure. (TIF) [file pgen.1005518.s002.tif]

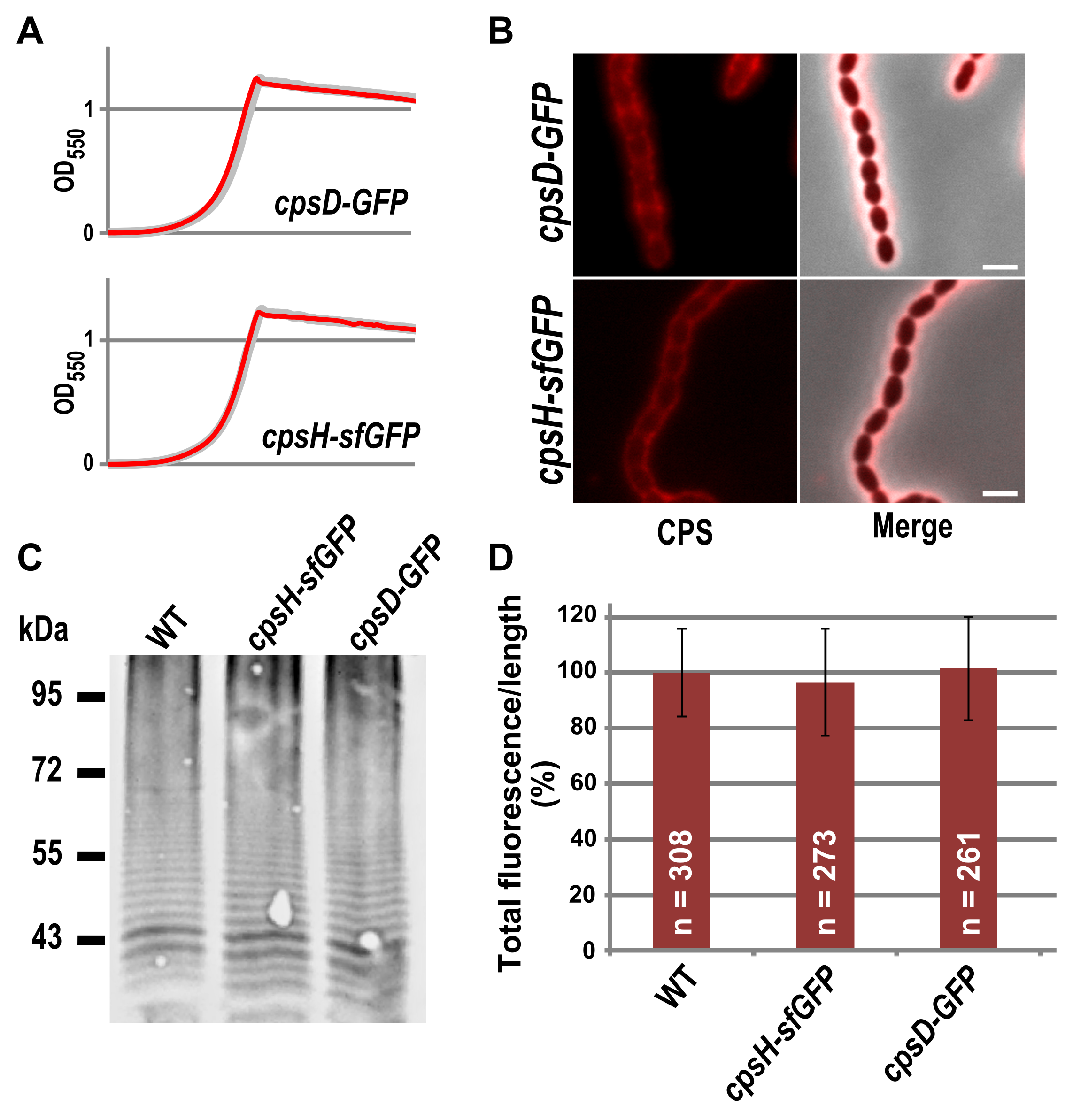

Supplement: S3 Fig — (A) Growth curves of WT strains expressing either CpsD-GFP (upper panel) or CpsH-sfGFP (lower panel) as the only source of CpsD or CpsH from their endogenous chromosomal locus grown in THY medium at 37°C. The OD550 was read automatically every 10 min. (B) Detection of CPS in living cpsD-GFP and cpsH-sfGFP cells. CPS were immunodetected with a rabbit anti-serotype 2 CPS polyclonal antibody. CPS fluorescent signal (red, left panels) and overlays (right panels) between phase contrast and CPS fluorescence images are shown. Scale bar, 2 μm. (C) Detection of cell-associated CPS produced by WT, cpsD-GFP and cpsH-sfGFP strains. The immunoblot was probed with a rabbit anti-serotype 2 CPS polyclonal antibody. (D) Quantification of the total CPS fluorescent signal in living WT, cpsD-GFP and cpsH-sfGFP cells. n indicates the number of cells analyzed and standard deviation from the fluorescence of the n cells is indicated with error bars. (TIF) [file pgen.1005518.s003.tif]

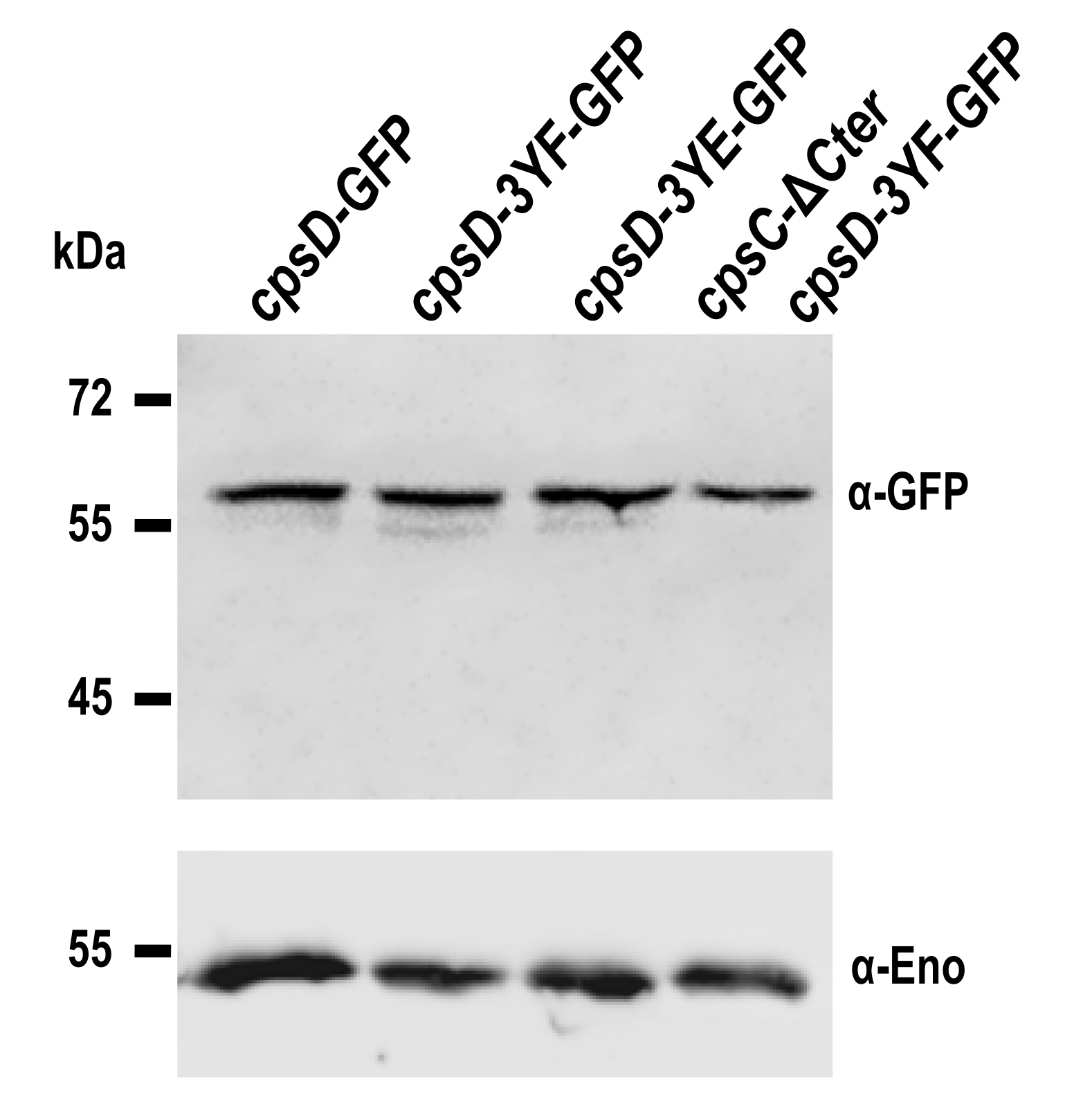

Supplement: S4 Fig — Expression of CpsD-GFP, CpsD-3YF-GFP, CpsD-3YE-GFP in WT cells and CpsD-3YF-GFP in cpsC-ΔCter cells. WT or mutated CpsD-GFP was detected using anti-GFP antibodies (α-GFP). To estimate the relative quantity of CpsD-GFP in crude extract and to compare the lanes, we used the enolase as an internal standard (α-Eno). The enolase was detected using specific antibodies [32] and is presented in the lower panel. (TIF) [file pgen.1005518.s004.tif]

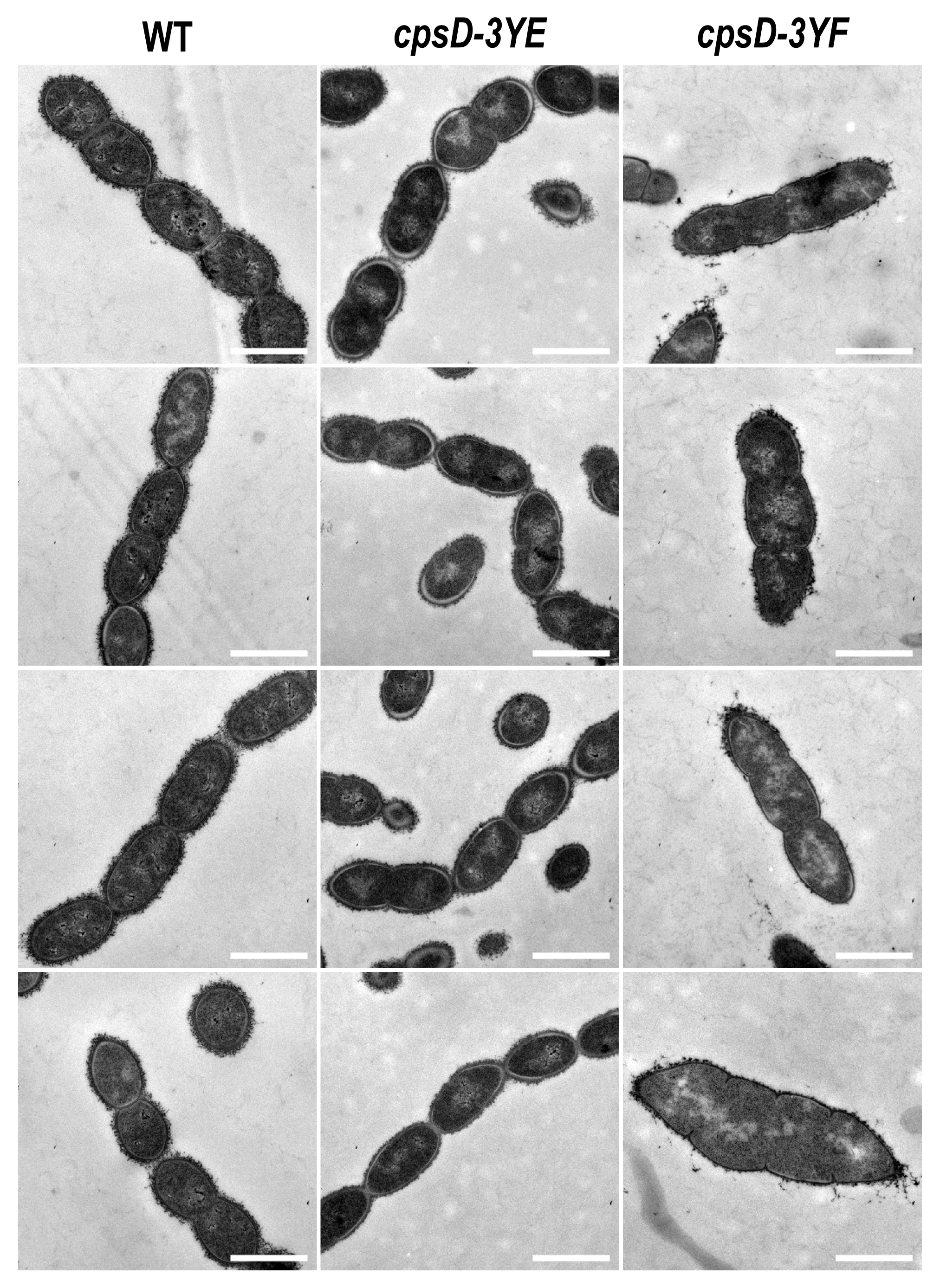

Supplement: S5 Fig — Transmission electron micrographs of WT (left column), cpsD-3YE (middle column) and cpsD-3YF (right column) strains. Scale bar, 1 μm. (TIF) [file pgen.1005518.s005.tif]

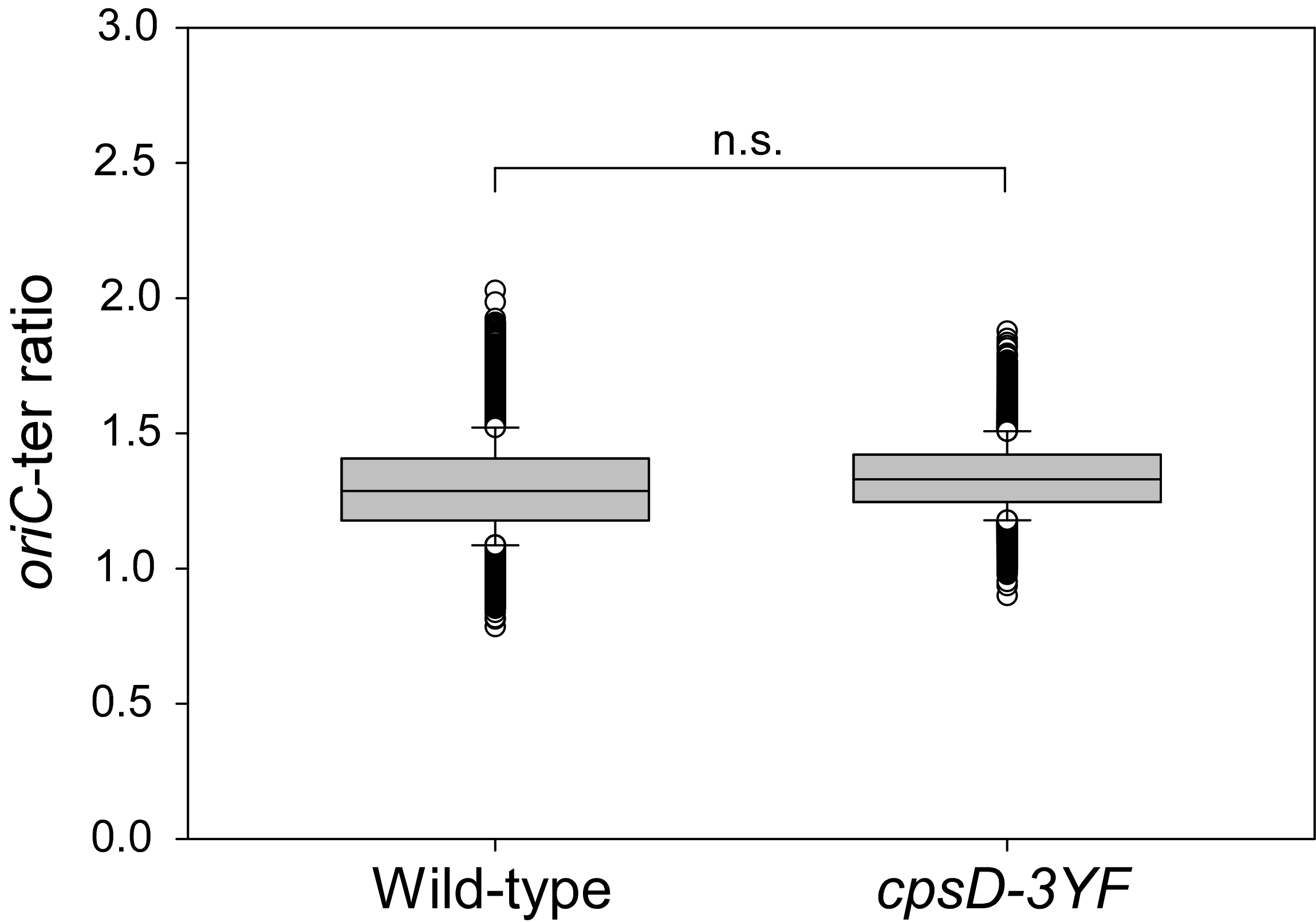

Supplement: S6 Fig — The CpsD-3YF mutation does not lead to differences in origin-to-terminus ratio. WT and CpsD-3YF were grown to OD600 = 0.15 for isolation of genomic DNA. The box-plots show the origin-to-terminus ratio as determined by qPCR. The data were analyzed by Monte Carlo simulations. There are no significant difference between the wild-type and the CpsD-3YF strain (p-value > 0.05). Whiskers represent the 10th and 90th percentiles, and the circles represent the outliers from Monte Carlo simulation data. qPCR, data analysis and the statistical test was done as described previously in [38]. (TIF) [file pgen.1005518.s006.tif]

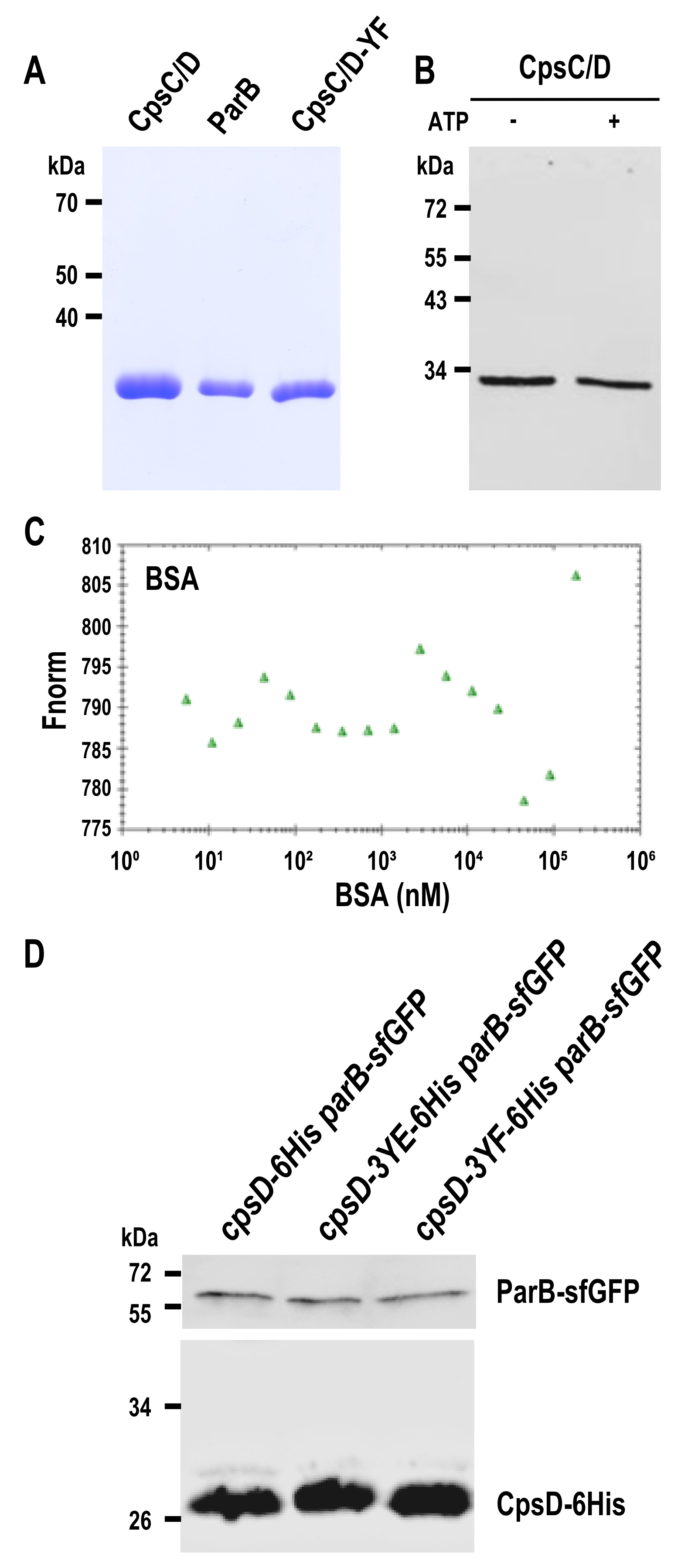

Supplement: S7 Fig — (A) Purification of ParB and chimeras CpsC/D and CpsC/D-YF. Proteins were overproduced in E. coli BL21 as 6his-tagged fusion proteins. After purification using a Ni-NTA agarose resin, they were analyzed by SDS-PAGE and coomassie blue staining. (B) Autophosphorylation of the chimera CpsC/D. 0.1 μg of purified CpsC/D from E. coli cells were incubated in the presence (+) or absence (-) of 5 mM ATP for 30 min at 37°C and analyzed by SDS-PAGE and electro-transferred onto a PVDF membrane. CpsD phosphorylation was then immunodetected using mouse anti-phosphotyrosine monoclonal antibody PY-20. (C) Affinity measurements by Microscale Thermophoresis of labeled ParB binding to increasing concentrations of BSA. No binding could be detected. (D) Expression level of the different forms of CpsD-6His and ParB before co-immunoprecipitation in cpsD-6His parB-sfGFP (lane 1), cpsD-3YE-6His parB-sfGFP (lane2) and cpsD-3YF-6His parB-sfGFP (lane 3) cells. (TIF) [file pgen.1005518.s007.tif]

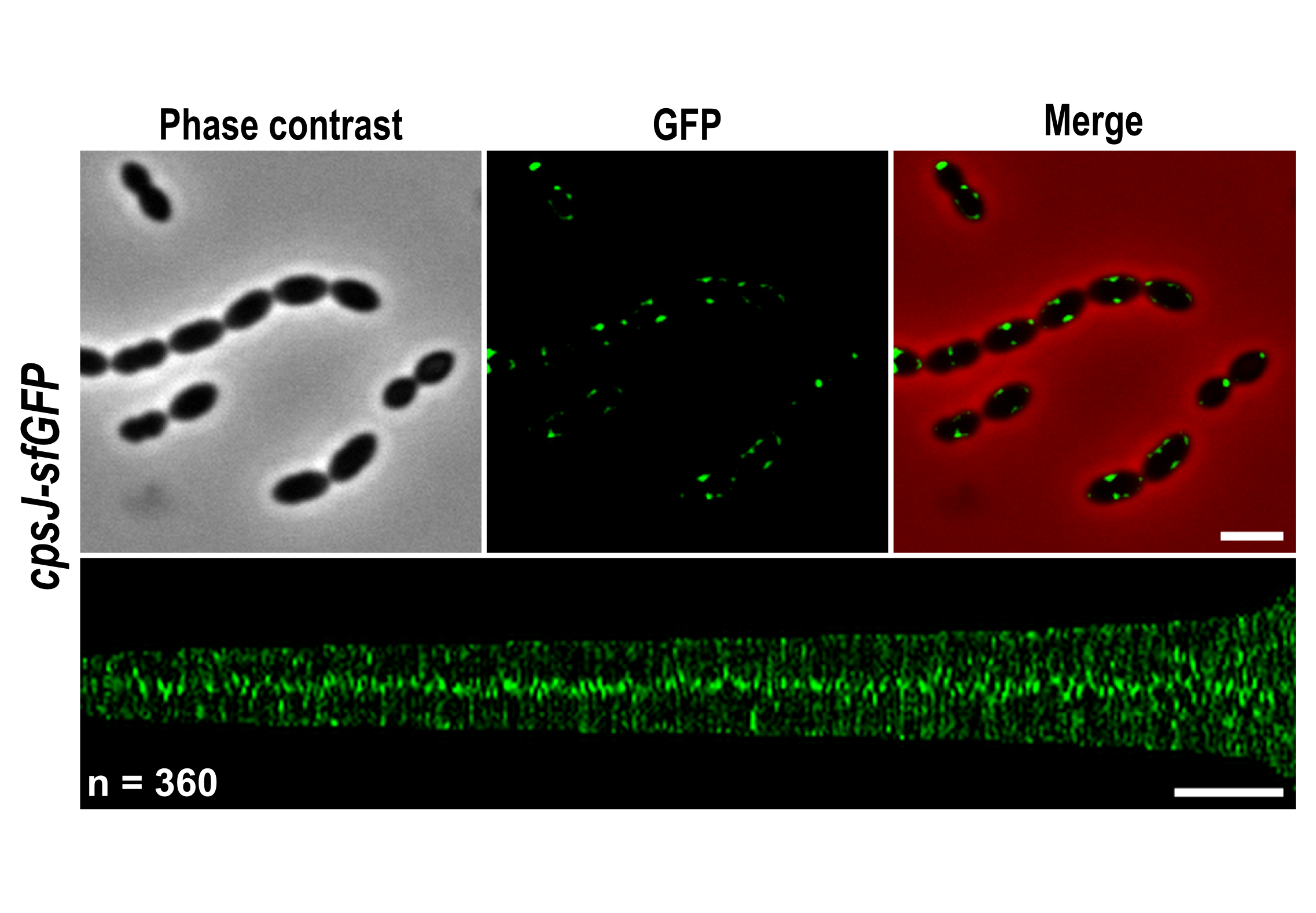

Supplement: S8 Fig — Phase contrast (left), GFP fluorescent signal (middle) and overlays (right) between phase contrast (red) and GFP (green) images are shown. The map of CpsJ-sfGFP fluorescence profiles of 360 cells sorted according to their length is presented. The total integrated fluorescence of each cell is plotted as function of its cell length (y-axes) and all cells are plotted with increasing cell length from left to right (x-axes). Scale bar, 2 μm. (TIF) [file pgen.1005518.s008.tif]

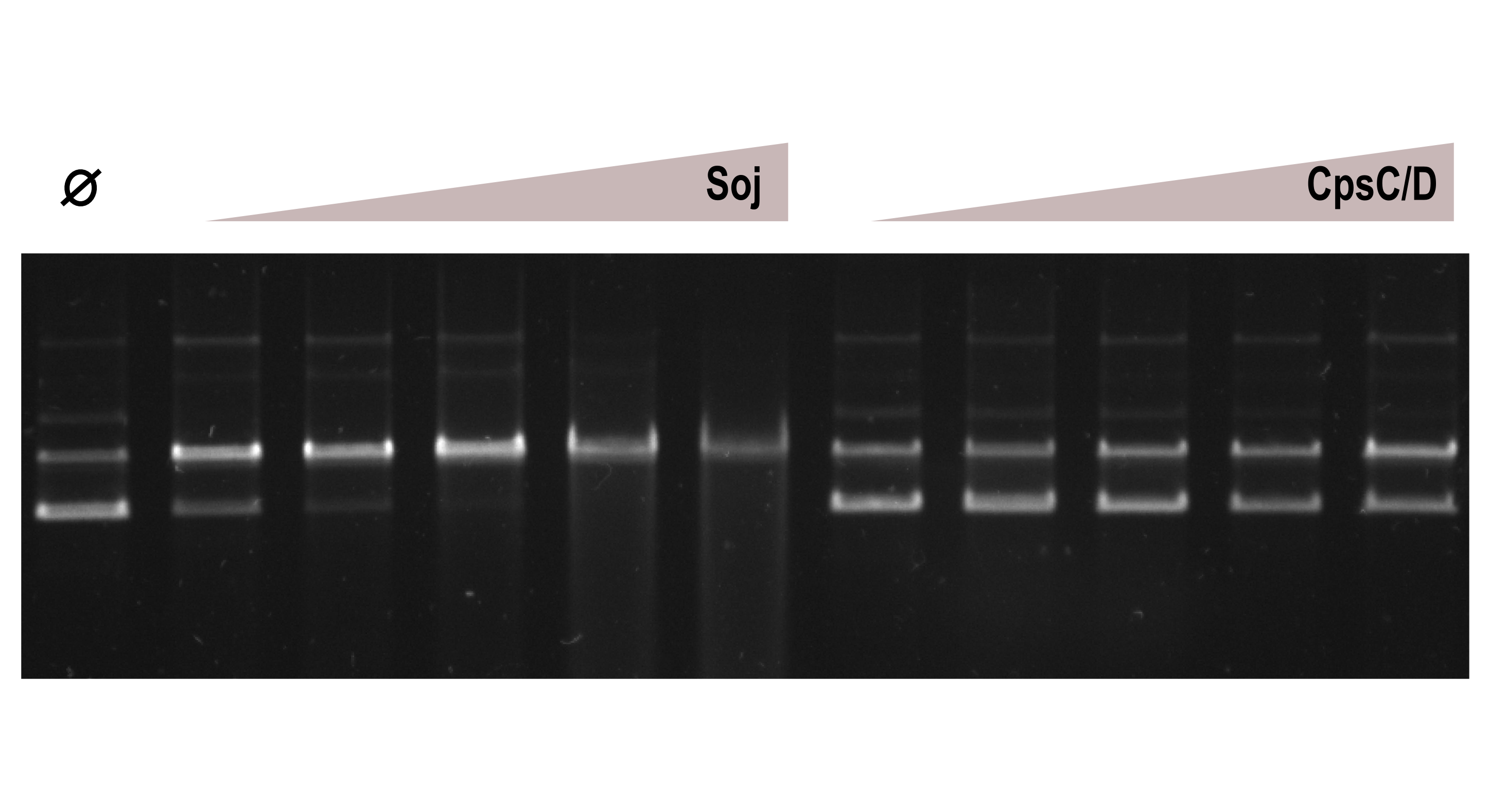

Supplement: S9 Fig — Soj from Bacillus subtilis was purified as described in [76]. Increasing amounts of Soj and CpsC/D were incubated with pUC18 DNA (20 fmol) in the presence of 1 mM ATP and run on a 0,6% agarose gel as previously described for Soj [76, 77]. Protein concentrations were as follow: Lane 1, no protein; Lane 2, SoJ 20 pmol; Lane 3, Soj 40 pmol; Lane 4, Soj 80 pmol; Lane 5, Soj 160 pmol; Lane 6, Soj 240 pmol; Lane 7, CpsC/D 20 pmol; lane 8, CpsC/D 40 pmol; Lane 9, CpsC/D 80 pmol; Lane 10, CpsC/D 160 pmol; Lane 11, CpsC/D 240 pmol. CpsC/D fails to bind DNA whereas Soj does. (TIF) [file pgen.1005518.s009.tif]
