# Supplementary material for: Autophosphorylation of the Bacterial Tyrosine-Kinase CpsD Connects Capsule Synthesis with the Cell Cycle in Streptococcus pneumoniae
Source: PLoS Genet. 2015 Sep 17;11(9):e1005518. doi: 10.1371/journal.pgen.1005518 (PMC4574921; doi:10.1371/journal.pgen.1005518)
Supplement: S1 Table — (PDF) [file pgen.1005518.s011.pdf]

S1 Table: Strains and plasmids used in this study.

Strain	Genotype and description	Reference
***S. pneumoniae* strains**		
D39	virulent strain	Gift from JP Claverys, Toulouse, France
Δ*cps*	D39 Δ*cps*	
R1226	R800 *rpsL1*, *cibABC*::*kan-rpsL*; Kan^R, Str^S	
D39 P$_M$	D39 with the P$_M$ promoter at the *amiF*/*treR* locus	Guiral *et al*., 2006
R800 *ftsZ-GFP*	R800 *rpsL1*, *ftsZ*::*ftsZ-gfp*; Str^R	Fleurie *et al*., 2014
D39 *hlpA-RFP*	D39, *hlpA::hlpA-rfp-cam*; Cam^R	Kjos and Veening, 2014
WT	D39 *rpsL::rpsL1*; Str^R	This study
Δ*cpsD*	*D39 rpsL::rpsL1;* Δ*cpsD*; Str^R	This study
Δ*cpsD* P$_M$-*cpsD*	*D39 rpsL::rpsL1;* Δ*cpsD;* P$_M$-*cpsD; Str*^{R}*, Tet*^{R}	This study
cpsD-3YE	*D39 rpsL::rpsL1; cpsD::cpsD-3YE*; Str^R	This study
cpsD-3YF	*D39 rpsL::rpsL1; cpsD::cpsD-3YF*; Str^R	This study
cpsD-GFP	*D39 rpsL::rpsL1; cpsD::cpsD-gfp*; Str^R	This study
cpsD-3YE-GFP	*D39 rpsL::rpsL1; cpsD::cpsD-3YE-gfp*; Str^R	This study
cpsD-3YF-GFP	*D39 rpsL::rpsL1; cpsD::cpsD-3YF-gfp*; Str^R	This study
cpsD-RFP	*D39 rpsL::rpsL1; cpsD::cpsD-rfp*; Str^R	This study
cpsD-6His	*D39 rpsL::rpsL1; cpsD::cpsD-6His*; Str^R	This study
cpsD-3YE-6His	*D39 rpsL::rpsL1; cpsD::cpsD-3YE-6His*; Str^R	This study
cpsD-3YF-6His	*D39 rpsL::rpsL1; cpsD::cpsD-3YF-6His*; Str^R	This study
*cpsC-*Δ*Cter*	*D39 rpsL::rpsL1; cpsC::cpsC-*Δ*Cter*; Str^R	This study
*cpsC-*Δ*Cter* P$_M$-*cpsC*	*D39 rpsL::rpsL1; cpsC::cpsC-*Δ*Cter;* P$_M$-*cpsC; Str*^{R}, *Tet*^{R}	This study
*cpsC-*Δ*Cter cpsD-GFP*	*D39 rpsL::rpsL1; cpsC::cpsC-*Δ*Cter; cpsD::cpsD-gfp*; Str^R	This study
parB-sfGFP	*D39 rpsL::rpsL1; parB::parB-sfgfp-spc*; Str^R, Spc^R	Minnen *et al*., 2011
cpsD-RFP parB-sfGFP	*D39 rpsL::rpsL1; cpsD::cpsD-rfp; parB::parB-sfgfp-spc*; Str^R, Spc^R	This study
cpsD-6His parB-sfGFP	*D39 rpsL::rpsL1; cpsD::cpsD-6His; parB::parB-sfgfp-spc*; Str^R, Spc^R	This study
cpsD-3YE-6His parB-sfGFP	*D39 rpsL::rpsL1; cpsD::cpsD-3YE-6His; parB::parB-sfgfp-spc*; Str^R, Spc^R	This study
cpsD-3YF-6His parB-sfGFP	*D39 rpsL::rpsL1; cpsD::cpsD-3YF-6His; parB::parB-sfgfp-spc*; Str^R, Spc^R	This study
ftsZ-GFP	*D39 rpsL::rpsL1; ftsZ::ftsZ-gfp*; Str^R	This study
cpsD-3YE ftsZ-GFP	*D39 rpsL::rpsL1; cpsD::cpsD-3YE; ftsZ::ftsZ-gfp*; Str^R	This study
cpsD-3YF ftsZ-GFP	*D39 rpsL::rpsL1; cpsD::cpsD-3YF; ftsZ::ftsZ-gfp*; Str^R	This study
cpsH-sfGFP	*D39 rpsL::rpsL1; cpsH::cpsH-sfgfp*; Str^R	This study
Δ*cpsD cpsH-sfGFP*	*D39 rpsL::rpsL1;* Δ*cpsD; cpsH::cpsH-sfgfp*; Str^R	This study
*cpsC-*Δ*Cter cpsH-sfGFP*	*D39 rpsL::rpsL1; cpsC::cpsC-*Δ*Cter; cpsH::cpsH-sfgfp*; Str^R	This study
cpsD-3YE cpsH-sfGFP	*D39 rpsL::rpsL1; cpsD::cpsD-3YE; cpsH::cpsH-sfgfp*; Str^R	This study
cpsD-3YF cpsH-sfGFP	*D39 rpsL::rpsL1; cpsD::cpsD-3YF; cpsH::cpsH-sfgfp*; Str^R	This study
hlpA-RFP	*D39 rpsL::rpsL1; hlpA::hlpA-rfp-cam*; Str^R, Cam^R	This study
cpsD-3YE hlpA-RFP	*D39 rpsL::rpsL1; cpsD::cpsD-3YE; hlpA::hlpA-rfp-cam*; Str^R, Cam^R	This study
cpsD-3YF hlpA-RFP	*D39 rpsL::rpsL1; cpsD::cpsD-3YF; hlpA::hlpA-rfp-cam*; Str^R, Cam^R	This study
hlpA-RFP parB-sfGFP	*D39 rpsL::rpsL1; hlpA::hlpA-rfp-cam; parB::parB-sfgfp-spc*; Str^R, Cam^R, Spc^R	This study
cpsD-3YE hlpA-RFP parB-sfGFP	*D39 rpsL::rpsL1; cpsD::cpsD-3YE; hlpA::hlpA-rfp-cam*; *parB::parB-sfgfp-spc*; Str^{R,} Cam^R, Spc^R	This study
cpsD-3YF hlpA-RFP parB-sfGFP	*D39 rpsL::rpsL1; cpsD::cpsD-3YF; hlpA::hlpA-rfp-cam*; *parB::parB-sfgfp-spc*; Str^R, Cam^R, Spc^R	This study
***E. coli* strains**		
XL1-Blue	*sup*E44 *hsd*R17 *rec*A1 *end*A1 *gyr*A46 *thi rel*A1 lac$^-$ F′[*pro*AB$^+$ *lac*I^q *lac*ZΔM15 Tn10 (Tc^R)]; Tet^R	Bullock et al., 1987
BL21(DE3)	F- *ompT gal dcm lon hsd*S$_B$(r$_B$- m$_B$-) λ(DE3 [*lac*I *lac*UV5-T7 gene 1 *ind*1 *sam*7 *nin*5])	Studier and Moffatt, 1986
**Plasmids**		
pT7.7	pT7.7 derivative, encoding a His-tag for C-terminal fusions; Amp^R	Cortay *et al*., 1994
pT7.7 *parB*	pT7.7 derivative, encoding ParB, from Met1 to Lys252; Amp^R	This study
pT7.7 *soj*	pT7.7 derivative, encoding Soj, from Met1 to Gly253; Amp^R	This study
pQE30	pQE30 derivative, encoding a His-tag for N-terminal fusions; Amp^R	Qiagen
pQE30-*cpsC*/*D* TIGR4	pQE30 derivative, encoding CpsD, from Met1 to Lys227, fused to the C-terminal part of CpsC, from Leu200 to Lys230; Amp^R	This study
pGBDU-C1	pGBDU derivative, encoding binding domain of Gal4 for N-terminal fusions; Amp^R, *ura3*	James *et al*., 1996
pGBDU-C1-*cpsD*	pGBDU derivative, encoding CpsD, from Met1 to Lys226; Amp^R, *ura3*	This study
pGBDU-C1-*cpsC*	pGBDU derivative, encoding CpsC, from Met1 to Lys230; Amp^R, *ura3*	This study
pGBDU-C1-*cpsC-*Δ*Cter*	pGBDU derivative, encoding CpsC, from Met1 to Phe201; Amp^R, *ura3*	This study
pGAD-C1	pGAD derivative, encoding activation domain of Gal4 for N-terminal fusions; Amp^R, *leu2*	James *et al*., 1996
pGAD-C1-*cpsD*	pGAD derivative, encoding CpsD, from Met1 to Lys226; Amp^R, *leu2*	This study
pGAD-C1-*cpsC*	pGAD derivative, encoding CpsC, from Met1 to Lys230; Amp^R, *leu2*	This study
pGAD-C1-*cpsC-*Δ*Cter*	pGAD derivative, encoding CpsC, from Met1 to Phe201; Amp^R, *leu2*	This study
pHK096	pJWV25 derivative, containing the *rfp* gene, from Met1 to Leu233; Amp^R	Beilharz *et al*., 2012
pUC57-*gfp*	pUC57 derivative, encoding the *gfp* gene, from Met1 to Lys239, Amp^R	Martin *et al*., 2010
pET21-*sfgfp*	pET21 derivative, encoding the *sfgfp* gene, from Met1 to Lys237,Amp^R	Dinh and Bernhardt, 2011
