# Supplementary material for: Autophosphorylation of the Bacterial Tyrosine-Kinase CpsD Connects Capsule Synthesis with the Cell Cycle in Streptococcus pneumoniae
Source: PLoS Genet. 2015 Sep 17;11(9):e1005518. doi: 10.1371/journal.pgen.1005518 (PMC4574921; doi:10.1371/journal.pgen.1005518)
Supplement: S2 Table — (PDF) [file pgen.1005518.s012.pdf]

S2 Table: Primers used in this study.

Purpose	Gene or plasmid	Name[a]	Sequence 5'-3'[b], gene, position[c]
1 - Construction of *S. pneumoniae* strains	*kan-rpsL*	A (+)	CCGTTTGATTTTTAATGGATAATG, Janus cassette of strain R1226
		B (-)	AGAGACCTGGGCCCCTTTCC, Janus cassette of strain R1226
	gfp	C (+)	AAACTAGACATCGAGTTCCTGCAGATGATTTCTAAAGGTGAAGAATTG, *gfp*, +1
		D (-)	TTATTTATACAATTCATCCATACC, *gfp*, +720
	rfp	E (+)	CTAGACATCGAGTTCCTGCAGATGAAACATCTTACCGGTTC, *rfp*, +1
		F (-)	CAATTTACTAGGCAAATCACAATAAC, *rfp*, +699
	sfgfp	G (+)	AAACTAGACATCGAGTTCCTGCAGTCTAAAGGTGAAGAACTGTTC, *sfgfp*, +1
		H (-)	TTATTTGTAGAGCTCATCCATGCCG, *sfgfp*, +714
	P_M	I (+)	ACCCTCATGAATTCTCAGGCGGT, upstream of insertion site
		J (-)	GGTAGCACCTCGTGTGTTAAAATAATGG, upstream of insertion site
		K (+)	GGATCCGTTTGATTTTTAATGGATAATG, downstream of insertion site
		L (-)	ATGAAGAAATACCAACAATTATTTAAGCAAATCC downstream of insertion site
	cpsD	1 (+)	TTGTGACAGGTGCGGGGGC, upstream of *cpsD*, -596
		2 (-)	TCAGCCCTACCAATGCACCC, downstream of *cpsD*, +1518
		3 (-)	**CATTATCCATTAAAAATCAAACGG**AACTTCCTCCTATTTCATTTTGTCC, upstream of *cpsD*, -1
		4 (+)	**GGAAAGGGGCCCAGGTCTCT**GTCGGGGGATAGAGATGAATGG, downstream of *cpsD*, +682
		5 (-)	**CATTCATCTCTATCCCCCGAC**AACTTCCTCCTATTTCATTTTGTCC, upstream of *cpsD*, -1
		6 (+)	GTCGGGGGATAGAGATGAATG, downstream of *cpsD*, +682
		7 (-)	CTGCAGGAA**CTCGATGTCTAGTTTTTTTTTATTTTTCCC**GTAATCTCCATAAG, downstream of *cpsD*, +679
		8 (+)	GGTATGGATGAATTGTATAAATAAGTCGGGGGATAGAGATGAATGG, downstream of *cpsD*, +682
		8' (+)	GTTATTGTGATTTGCCTAGTAAATTGTAAGTCGGGGGATAGAGATGAATGGAAAAACAG, downstream of *cpsD*, +682
		9 (+)	GGGAAAAATAAAAAATAGGTCGGGGGATAG, downstream of *cpsD*, +664
		10 (-)	**CTATCCCCCGACCTATTTTTTATTTTTCCC**cTcATCTCCcTcAGAACCcTcTTTGTCTACTGAAG, downstream of *cpsD*, +663
		10' (-)	**CTATCCCCCGACCTATTTTTTATTTTTCCC**GaAATCTCCAaAAGAACCGaATTTGTCTACTGAAG, downstream of *cpsD*, +663
		11 (+)	GGGAAAAATAAAAAAAAACTAGACATCGAG, dowstream of *cpsD*, +664
		12 (-)	CTCGATGTCTAGTTTTTTTTTATTTTTCCCcTcATCTCCcTcAGAACCcTcTTTGTCTACTGAAG, dowstream of *cpsD*, +663
		12' (-)	CTCGATGTCTAGTTTTTTTTTATTTTTCCCGaAATCTCCAaAAGAACCGaATTTGTCTACTGAAG, dowstream of *cpsD*, +663
		13 (+)	**CCATTATTTTAACACACGAGGTGCTACC**ATGCCAACGTTAGAAATCTCAC, *cpsD*, +1
		14 (-)	**CATTATCCATTAAAAATCAAACGGATCC**CTATTTTTTATTTTTCCCGTAATCTCC, *cpsD*, +681
		6His (+)	**GGCCACCATCATCATCATCATCACGGCTAA**GTCGGGGGATAGAGATGAATGG, dowstream of *cpsD*, +682
		6His (-)	**TTAGCCGTGATGATGATGATGATGGTGGCC**TTTTTTATTTTTCCCGTAATCTCC, dowstream of *cpsD*, +679
		6His' (-)	**TTAGCCGTGATGATGATGATGATGGTGGCC**TTTTTTATTTTTCCCcTcATCTCCc, dowstream of *cpsD*, +679
		6His" (-)	**TTAGCCGTGATGATGATGATGATGGTGGCC**TTTTTTATTTTTCCCGaAATCTCC, dowstream of *cpsD*, +679
	cpsC	1 (+)	ATAGACGTCCATTCGCATATCG, upstream of *cpsC*, -735
		2 (-)	CTCCCAAAAACGGCTTCCCTG, downstream of *cpsC*, +1312
		3 (-)	**CATTATCCATTAAAAATCAAACGG**TTCTCCTAAATTAGTTGATCCATTAC, upstream of *cpsC*, -1
		4 (+)	**GGAAAGGGGCCCAGGTCTCT**AGGAGGAAGTTATGCCAACG, downstream of *cpsC*, +695
		5 (-)	**CTAACGTTGGCATAACTTCCTC**CTAGAACAACTCAATCAAAAGAAC, downstream of *cpsC*, +586
		6 (+)	GAGGAAGTTATGCCAACGTTAG, downstream of *cpsC*, +697
		13 (+)	**CCATTATTTTAACACACGAGGTGCTACC**ATGAAAGAACAAAACACGATAGAAATCG, *cpsC*, +1
		14 (-)	**CATTATCCATTAAAAATCAAACGGATCCCTATTTCATTTTGTCCAAATCTGGAAC**, *cpsC*, +693
	cpsH	1 (+)	GAGGCATCGTATCGCTTGC, upstream of *cpsH*, -704
		2 (-)	CTAAATCAACCTGCCCTTTTCC, downstream of *cpsH*, +1963
		3 (-)	**CATTATCCATTAAAAATCAAACGG**TTATTTTTCTTGCTTAGTCAATCTCATTC, downstream of *cpsH*, +1164
		4 (+)	**GGAAAGGGGCCCAGGTCTCT**GTGCATTTATAGGAATGAGATTGAC, downstream of *cpsH*, +1124
		7 (-)	CTGCAGGAACTCGATGTCTAGTTTTTTTTCTTGCTTAGTCAATCTCATTC, downstream of *cpsH*, +1161
		8 (+)	CGGCATGGATGAGCTCTACAAATAAAAATGGATGGGGAAATTCAAGG, downstream of *cpsH*, +1165
	ftsZ	1 (+)	CCTATCCGCCTCTTGCAAGC, upstream of *ftsZ*, -607
		2 (-)	CTTTTAAAGACATGGTTCTCTCCTAC, downstream of *ftsZ*, +1958
	parB	1 (+)	CTGACACTTTCTCTGATATTGC, upstream of *parB*, -801
		2 (-)	GGGATATATTTAACACGCGCATTAGG, downstream of *parB*, +1525
	hlpA	1 (+)	AACAAGTCAGCCACCTGTAG, upstream of *hlpA*, -763
		2 (-)	CGTGGCTGACGATAATGAGG, downstream of *hlpA*, +1353
2 - Construction of plasmids for protein overexpression in *E. coli*	pQE30-*cpsC*$_{cyto}$/*cpsD* T4	I (+)	TAT*GGATCC*CTTTTGGATACTCGTGTGAAAC, *cpsC*, +658 (*BamHI*)
		II (-)	TAT*AAGCTT*TTATTTTTTACCATAATTTCCATAGG, *cpsD*, +684 (*HindIII*)
		III (-)	**GTGCTATTTCTAATGTCGGCAT**TTTCAACTTACCCAAGTTTGG, *cpsC*, +693
		IV (+)	ATGCCGACATTAGAAATAGCAC, *cpsD*, +1
	pT7.7-*parB*	I (+)	TAT*CATATG*GAAAAATTTGAAATGATTTCTATC, *parB*, +1 (*NdeI*)
		II (-)	TAT*CTGCAG*TTTCAGGCTGTTGATAATTCTACT, *parB*, +756 (*PstI*)
	pT7.7-*soj*	I (+)	TAT*CATAT*GGGAAAAATCATAGCAATTACGAAC, *soj* +1 (*NdeI*)
		II (-)	TAT*CTGCAG*GCCATTCGCAGCCACTTCCTTTGC, *soj* +759 (*PstI*)
3 - Construction of plasmids for yeast two hybrid assay	*cpsC*	I (+)	TAT*GAATTC*AAAGAACAAAACACGATAGAAATC, *cpsC*, +1 (*EcoRI*)
		II (-)	TAT*CTGCAG*CTATTTCATTTTGTCCAAATCTGG, *cpsC*, +696 (*PstI*)
		II' (-)	TAT*CTGCAG*TTACAACTCAATCAAAAGAACAGC, *cpsC*, +603 (*PstI*)
	cpsD	I (+)	TAT*GAATTC*CCAACGTTAGAAATCTCACAGGCA, *cpsD*, +1 (*EcoRI*)
		II (-)	TAT*CTGCAG*CTATTTTTTATTTTTCCCGTAATC, *cpsD*, +681 (*PstI*)

[a] Forward and reverse primers are represented by plus (+) or minus (-), respectively.

[b] The primers 1 and 2 encode for upstream and downstream sequences of our genes of interest, respectively. DNA sequences shown in bold in primers 3 and 4 are complementary to the 5'-end (primer A) and the 3'-end (primer B) of the *kan-rpsL* sequence, respectively. Sequences shown in bold in primers 5 are complementary to the primer 6. Sequences underlined in primers 7 are complementary to sequences underlined in primers C, E or G and code for a linker inserted between the protein of interest and the GFP, RFP or sfGFP. Sequences underlined in primers 8 and 8' are complementary to the sequence underlined in primers D, F or H and the 3'-end of the *gfp*, *rfp* or *sfgfp* sequences. Sequences shown in bold in primers 10 and 10' are complementary to the primer 9. Sequences shown in bold in primers 7, 12 and 12' are complementary to the primer 11. Sequences shown in bold in primers 13 and 14 are complementary to primers J and K, respectively. Sequence shown in bold in primer 6His (+) is complementary to sequences shown in bold in primers 6His (-), and code for a 6-Histidines tag. The primers I and II encode for 5' and 3' extremities of our genes of interest, respectively and are used for their insertion in pGAD-C1 and pGBDU-C1. The sequence shown in bold in the primer III is complementary to the primer IV. The primer II' is necessary to construct *cpsC*-Δ*Cter*. Mutated bases are in lowercase. Restriction sites are italicized and the corresponding restriction enzymes are indicated in brackets.

[c] - and + indicate respectively upstream and downstream positions relative to the ATG codon of the corresponding gene.
